# Applications of Information Theory in Solar and Space Physics

**DOI:** 10.3390/e21020140

**Published:** 2019-02-01

**Authors:** Simon Wing, Jay R. Johnson

**Affiliations:** 1 Applied Physics Laboratory, the Johns Hopkins University, Laurel, MD 20723-6099, USA; 2 Andrews University, Berrien Springs, MI 49104, USA

**Keywords:** information theory, radiation belts, solar dynamo, mutual information, conditional mutual information, transfer entropy, solar cycle, sunspot number, solar wind drivers, geosynchronous orbit electron flux

## Abstract

Characterizing and modeling processes at the sun and space plasma in our solar system are difficult because the underlying physics is often complex, nonlinear, and not well understood. The drivers of a system are often nonlinearly correlated with one another, which makes it a challenge to understand the relative effects caused by each driver. However, entropy-based information theory can be a valuable tool that can be used to determine the information flow among various parameters, causalities, untangle the drivers, and provide observational constraints that can help guide the development of the theories and physics-based models. We review two examples of the applications of the information theoretic tools at the Sun and near-Earth space environment. In the first example, the solar wind drivers of radiation belt electrons are investigated using mutual information (MI), conditional mutual information (CMI), and transfer entropy (TE). As previously reported, radiation belt electron flux (*J_e_*) is anticorrelated with solar wind density (*n_sw_*) with a lag of 1 day. However, this lag time and anticorrelation can be attributed mainly to the *J_e_*(*t* + 2 days) correlation with solar wind velocity (*V_sw_*)(*t*) and *n_sw_*(*t* + 1 day) anticorrelation with *V_sw_*(*t*). Analyses of solar wind driving of the magnetosphere need to consider the large lag times, up to 3 days, in the (*V_sw_*, *n_sw_*) anticorrelation. Using CMI to remove the effects of *V_sw_*, the response of *J_e_* to *n_sw_* is 30% smaller and has a lag time <24 h, suggesting that the loss mechanism due to *n_sw_* or solar wind dynamic pressure has to start operating in <24 h. Nonstationarity in the system dynamics is investigated using windowed TE. The triangle distribution in *J_e_*(*t* + 2 days) vs. *V_sw_*(*t*) can be better understood with TE. In the second example, the previously identified causal parameters of the solar cycle in the Babcock–Leighton type model such as the solar polar field, meridional flow, polar faculae (proxy for polar field), and flux emergence are investigated using TE. The transfer of information from the polar field to the sunspot number (*SSN*) peaks at lag times of 3–4 years. Both the flux emergence and the meridional flow contribute to the polar field, but at different time scales. The polar fields from at least the last 3 cycles contain information about *SSN*.

## 1. Introduction

For many complex systems, modeling can be physically or computationally difficult. The coupled solar wind–magnetosphere system is nonlinear and complex. Successful predictions of phenomena such as magnetic storms or substorms have remained notoriously elusive, despite numerous attempts over several decades. It has been known for over a century that the Sun has cool regions of high magnetic flux density that known as sunspots. The number of sunspots exhibits an 11-year cycle, but predicting the sunspot number (*SSN*) has been largely unsuccessful. The dynamo that generates the sunspots is complex and nonlinear. There have been significant advances in physics-based modeling of solar, magnetospheric, and ionospheric processes, but the global computations are beyond present and/or near future computational capabilities without appropriate approximations. Empirical models that employ intuition assume a priori a dynamical framework that may or may not apply to the system. Moreover, care must be taken because it may be possible to fit the data by choosing enough free parameters at the expense of losing physical understanding or overfitting.

Dependency is a key discriminating statistic that is commonly used to understand how systems operate. The standard tool used to identify dependency is cross-correlation. Considering two variables, *x* and *y*, the correlation analysis essentially tries to fit the data to a 2D Gaussian cloud, where the nature of the correlation is determined by the slope and the strength of correlation is determined by the width of the cloud perpendicular to the slope. The correlational analyses are useful, fast, and simple. However, they cannot describe nonlinear relationships and usually cannot be used to establish causalities.

The entropy-based information theory can help identify nonlinearities in the system, information transfer between input and output parameters, and the lag response times. This nonparametric, statistics-based method is not constrained by the assumption of an underlying dynamics---rather the underlying (physics-based) dynamics is discovered by the approach and then ultimately utilized to improve predictions. Moreover, it can help untangle the input parameters that are correlated or anti-correlated with each other. It can also help modelers select input parameters and determine prediction horizon. The latter refers to how far ahead can one predict a variable. Hence, it can be a useful tool to study many complex systems. This approach should be considered complimentary to correlational analyses and to physics-based and empirical modeling approaches.

In the present paper, we review our recent work applying information theory to two complex systems: (1) solar wind–radiation belt system and (2) solar dynamo. The information theoretic tools that we use are mutual information [1,2], conditional mutual information [3], and transfer entropy [4].

The rest of paper is organized as follows. Section 2 describes the datasets and Section 3 summarizes briefly the information theoretic tools. Section 4 describes the application to solar wind–radiation belt system. Section 5 describes the solar dynamo. Section 6 presents the concluding remarks.

## 2. Data Set

The solar wind–radiation belt system study uses daily averages of MeV electron fluxes obtained from Energetic Sensor for Particles (ESP) [5] and Synchronous Orbit Particle Analyzer (SOPA) [6] on board of all seven Los Alamos National Laboratory (LANL) geosynchronous satellites from 22 Sep 1989 to 31 Dec 2009. The data and format description can be found at ftp://ftop.agu.org/apend/ja/2010ja015735. We only examine the fluxes of electrons with energy range of 1.8–3.5 MeVs. A detailed description of the dataset and its processing are given in [7]. The daily and hourly averaged solar wind data 1989–2009 come from OMNI dataset provided by NASA (http://omniweb.gsfc.nasa.gov/). The LANL and solar wind data are merged. The LANL dataset has 7187 data points (days of data), out of which, 6438 data points have simultaneous solar wind observations.

The solar dynamo study uses multiple datasets of the Sun and Earth. The *SSN* record 1749–2016 is provided by Sunspot Index and Long-term Solar Observations (SILSO) website at http://www.sidc.be/silso/datafiles. The meridional flow dataset 1986–2012 is derived from Mount Wilson Observatory (MWO) dopplergrams and magnetograms as described in [8] and is available at http://www.astro.ucla.edu/~ulrich/. The polar faculae dataset 1906–2014 is based on the consolidated data from MWO, Wilcox Solar Observatory (WSO), and Solar and Heliospheric Observatory (SOHO) Michelson Doppler Imager (MDI) [9]. This dataset is available from Solar Polar Fields Dataverse at https://dataverse.harvard.edu/dataverse/solardynamo. The polar field dataset 1967–2015 is derived from MWO and WSO photospheric field maps with the line of sight profile saturation corrections [10,11] (acquired from Yi-Ming Wang). The record of the *aa* index (1868–2010) is provided by the National Oceanic and Atmospheric Administration National Centers for Environmental Information (NOAA NCEI) website: ftp://ftp.ngdc.noaa.gov/STP/GEOMAGNETIC_DATA/ and 1869–2015 from International Service of Geomangetic Indices (ISGI) at http://sgi.unistra.fr/indices_aa.php. *aa* index is a geomagnetic activity index [12]. All the data analysis is performed at monthly resolution.

## 3. Mutual Information, Conditional Mutual Information, and Transfer Entropy

Information theory has been applied to problems in magnetospheric, ionospheric, and solar physics [13,14,15,16,17,18,19,20,21,22,23,24,25,26]. For example, mutual information and transfer entropy have been successfully used to examine the causal relationships among solar wind, storms, and substorms in the Earth’s magnetosphere [27,28]. Mutual information, conditional mutual information, and transfer entropy are briefly described below, but they are reviewed in [17].

Mutual information (MI) [1,2,29] between two variables, *x* and *y*, compares the uncertainty of measuring variables jointly with the uncertainty of measuring the two variables independently. The uncertainty is measured by Shannon entropy. In order to construct the entropies, it is necessary to obtain the probability distribution functions, which in this study are obtained from histograms of the data based on discretization of the variables (i.e., bins). Suppose that two variables, *x* and *y*, are binned so that they take on discrete values, $\hat{x}$ and $\hat{y}$, where

(1) $$x\;\in \;\left\{{\hat{x}}_{1},\;{\hat{x}}_{2},\;\cdots ,\;{\hat{x}}_{n}\right\}\;\equiv \;{ℵ}_{1};\;y\;\in \;\left\{{\hat{y}}_{1},\;{\hat{y}}_{2},\;\cdots ,\;{\hat{y}}_{n}\right\}\;\equiv \;{ℵ}_{2}$$

The variables may be thought of as letters in alphabets ℵ_1_ and ℵ_2_, which have *n* and *m* letters, respectively. The extracted data can be considered as sequences of letters. The entropy associated with each of the variables is defined as

(2) $$H\left(x\right)=\;-\;\sum_{{ℵ}_{1}}p\left(\hat{x}\right)\;\log p\left(\hat{x}\right)\;;\;H\left(y\right)=-\;\sum_{{ℵ}_{2}}p\left(\hat{y}\right)\;\log p\left(\hat{y}\right)$$

where *p*($\hat{x}$) is the probability of finding the letter $\hat{x}$ in the set of *x*-data and *p*($\hat{y}$) is the probability of finding letter $\hat{y}$ in the set of *y*-data. To examine the relationship between the variables, we extract the word combinations ($\hat{x}$, $\hat{y})$ from the dataset. The joint entropy is defined by

(3) $$H\left(x,\;y\right)=\;-\;\underset{{ℵ}_{1}{ℵ}_{2}}{\sum }p\left(\hat{x},\;\hat{y}\right)\;\log\;p\left(\hat{x},\;\hat{y}\right)$$

where $p\left(\hat{x},\;\hat{y}\right)$ is the probability of finding the word combination $\left(\hat{x},\;\hat{y}\right)$ in the set of (*x*, *y*) data. The mutual information [1,2,29] is then defined as

*MI (x, y) = H (x) + H (y) − H (x, y)* (4)

While MI is useful to identify nonlinear dependence between two variables, it is often useful to consider conditional dependency with respect to a conditioner variable *z* that takes on discrete values, $\hat{z}$ ∈ { *z_1_, z_2_, …z_n_*} ≡ ℵ_3_. The conditional mutual information [3]

(5) $$\mathrm{CMI}(x,y|z)=\sum_{{ℵ}_{1}{ℵ}_{2}{ℵ}_{3}}p\left(\hat{x},\;\hat{y},\;\hat{z}\right)log\frac{p\left(\hat{x},\;\hat{y}\;|\;\hat{z}\right)}{p\left(\hat{x}\;|\;\hat{z}\right)\;p\left(\hat{y}\;|\;\hat{z}\right)}=H(x,z)+H(y,z)-H(x,y,z)-H(z)$$

determines the mutual information between *x* and *y* given that *z* is known. In the case where *z* is unrelated, CMI*(x,y|z) = MI(x,y)*, but in the case that *x* or *y* is known based on *z*, then CMI*(x,y|z)* = 0. CMI therefore provides a way to determine how much additional information is known given another variable. CMI can be seen as a special case of the more general conditional redundancy that allows the variable *z* to be a vector [30].

A common method to establish causal relationships between two time series, e.g., [*x_t_*] and [*y_t_*], is to use a time-shifted correlation function

(6) $$r\left(\tau \right)=\frac{\langle x_{t}\;y_{t+\tau }\rangle -\langle x\rangle \;\langle y\rangle }{\sqrt{\langle x^{2}\rangle -\langle x^{2}\rangle }\;\sqrt{\langle y^{2}\rangle -\langle y^{2}\rangle }}$$

where *r* = correlation coefficient and *τ* = lag time. The results of this type of analysis may not be particularly clear when the correlation function has multiple peaks or there is not an obvious asymmetry. Additionally, correlational analysis only detects linear correlations. If the feedback involves nonlinear processes, its usefulness may be seriously limited.

Alternatively, time shifted mutual information, MI(*x*(*t*), *y*(*t* + *τ*)), can be used to detect causality in nonlinear systems, but this too suffers from the same problems as time-shifted correlation when it has multiple peaks and long range correlations.

A better choice for studying causality is the one-sided transfer entropy [4]

(7) $$TE_{x\to y}\left(\tau \right)\;=\;-\;\underset{{ℵ}_{1}{ℵ}_{2}}{\sum }p\left(y_{t+\tau },\;yp_{t},\;x_{t}\right)\;\log\left(\frac{p\left(y_{t+\tau }\;|\;yp_{t},\;x_{t}\right)}{p\left(y_{t+\tau }\;|\;yp_{t}\right)}\right)$$

where $yp_{t}=\;\left[y_{t},\;y_{t-\Delta },\;\cdots ,\;y_{t-k\Delta }\right]$, *k* + 1 = dimensionality of the system, and Δ= first minimum in MI[*y(t)*, *y(t − τ)*]. Transfer entropy (TE) can be considered as a specialized case of conditional mutual information:

(8) $$TE_{x\to y}\left(\tau \right)\;=\;CMI(y\left(t+\;\tau \right),x\left(t\right)|yp\left(t\right))$$

where yp(t)= [y(t), y(t− Δ),⋯,y(t−kΔ)]. The transfer entropy can be considered as a conditional mutual information that detects how much average information is contained in an input, *x*, about the next state of the system, *y*, that is not contained in the past history, *yp*, of the system [31]. In the absence of information flow from *x* to *y*, TE(*x* → *y*) vanishes. Also, unlike correlational analysis and mutual information, transfer entropy is directional, TE(*x* → *y*) ≠ TE(*y* → *x*). The transfer entropy accounts for static internal correlations, which can be used to determine whether *x* and *y* are driven by a common driver or whether *x* drives *y* or *y* drives *x*.

## 4. The Solar Wind–Radiation Belt System

The Earth’s radiation belts refer to a region in space that is populated by trapped energetic particles, electrons and ions. Typically, there are two radiation belts: inner belt and outer belt, but sometimes a third belt appears between the two belts [32]. The inner belt is located at equatorial distance approximately between 1.2 and 3 *R_E_* (*R_E_* = radius of the Earth ~6371 km) from the center of the Earth and is populated by electrons having energies of hundreds of keVs and ions having hundreds of MeVs. The outer belt is located at equatorial distance approximately between 4 and 8 *R_E_* and is mostly populated by electrons having energies ranging from a few hundred keVs to tens of MeVs. The present paper deals only with the outer radiation belt electron population.

These radiation belt electrons are often referred to as “killer electrons” because they can cause serious damage to satellites. For example, the radiation belt electrons with energies of a few MeVs or higher can penetrate deep into spacecraft components, while those with energies lower than one MeV can lodge on the surface of the spacecraft bodies, leading to devastating electrical discharges. Radiation belts are quite relevant to the studies of space weather.

The existence of radiation belt MeV electrons is usually explained by some acceleration mechanisms that can accelerate electrons from a few keVs to tens of MeVs. There have been several acceleration mechanisms proposed, but most studies generally suggest either local or global acceleration. In the local acceleration, the storm and substorm injects plasma sheet plasma into the inner-magnetosphere and accelerates low energy (e.g., a few keV) electrons to a few hundred keVs. Once in the inner-magnetosphere, electrons interact with locally grown ultra low frequency (ULF) waves [33,34,35,36,37,38], very low frequency (VLF) waves [39,40,41,42,43], or magnetosonic waves [44,45], which can energize electrons to MeV energy range. Solar wind velocity (*V_sw_*) may be tied to the local acceleration mechanism through substorm particle injections [46,47,48,49,50].

The global acceleration mechanism also invokes ULF waves for electron acceleration, but here the ULF waves are generated globally by Kelvin-Helmholtz instability (KHI) along the magnetopause flanks due to large velocity shear between solar wind and the magnetospheric plasma [51,52,53]. Indeed, studies have shown that *V_sw_* is a dominant, if not the most dominant, driver of relativistic electron fluxes at geosynchronous orbit (6.6 *R_E_*) [7,49,54,55,56,57,58].

**Figure 1:**
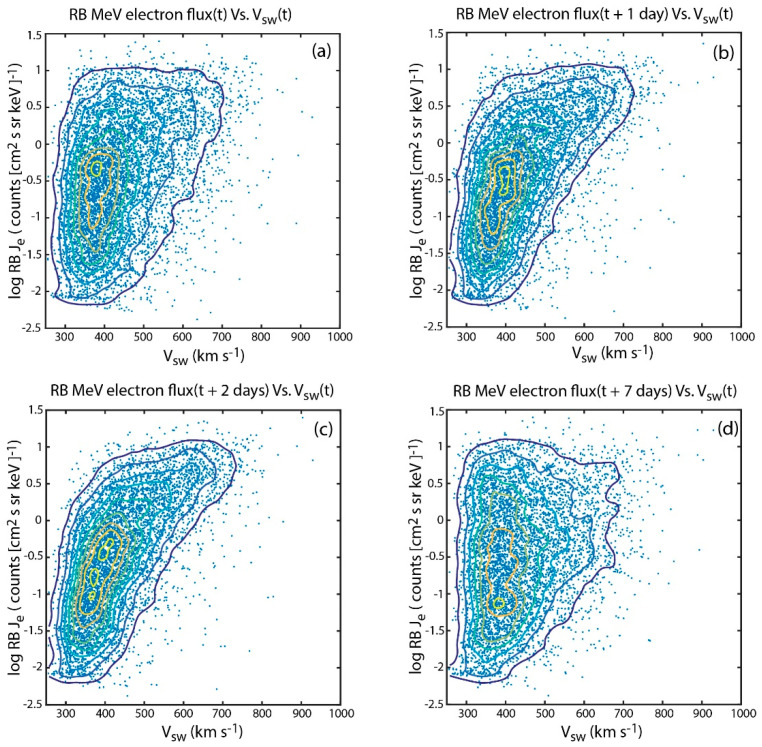
Scatter plots of log *J_e_*(*t* + *τ*) vs. *V_sw_*(*t*) for *τ* = 0, 1, 2, and 7 days in panels (**a**), (**b**), (**c**), and (**d**), respectively. The data points are overlain with density contours showing the nonlinear trends. The panels show that *J_e_* has dependence on *V_sw_* for *τ* = 0, 1, and 2 days and the dependence is strongest for *τ* = 2 days. (**d**) At large *τ*, e.g., *τ* = 7 day, *J_e_* dependence on *V_sw_* is very weak. This figure is essentially the same as Figure 9 in [7], except that no density contours are drawn and Figure 1d plots *τ* = 7 days instead of *τ* = 3 days. (from [23]).

Figure 1 plots the geosynchronous radiation belt MeV electron flux (*J_e_*) vs. *V_sw_* where *J_e_* is the relativisic electron flux. The figure shows log *J_e_*(*t* + *τ*) vs. *V_sw_*(*t*) for *τ* = 0, 1, 2, and 7 days. A few things are worth noting. First, the solar wind–radiation belt system is nonlinear and hence the standard linear correlational analysis would be inadequate. Second, the scatter plots in panel a–c look like a triangle, which is discussed in Section 4.2 using information theory. Third, the radiation belt *J_e_* is correlated with *V_sw_*. The best correlation can be found with *J_e_* with a two-day lag, but it is hard to see this in the scatter plot in Figure 1.

**Figure 2:**
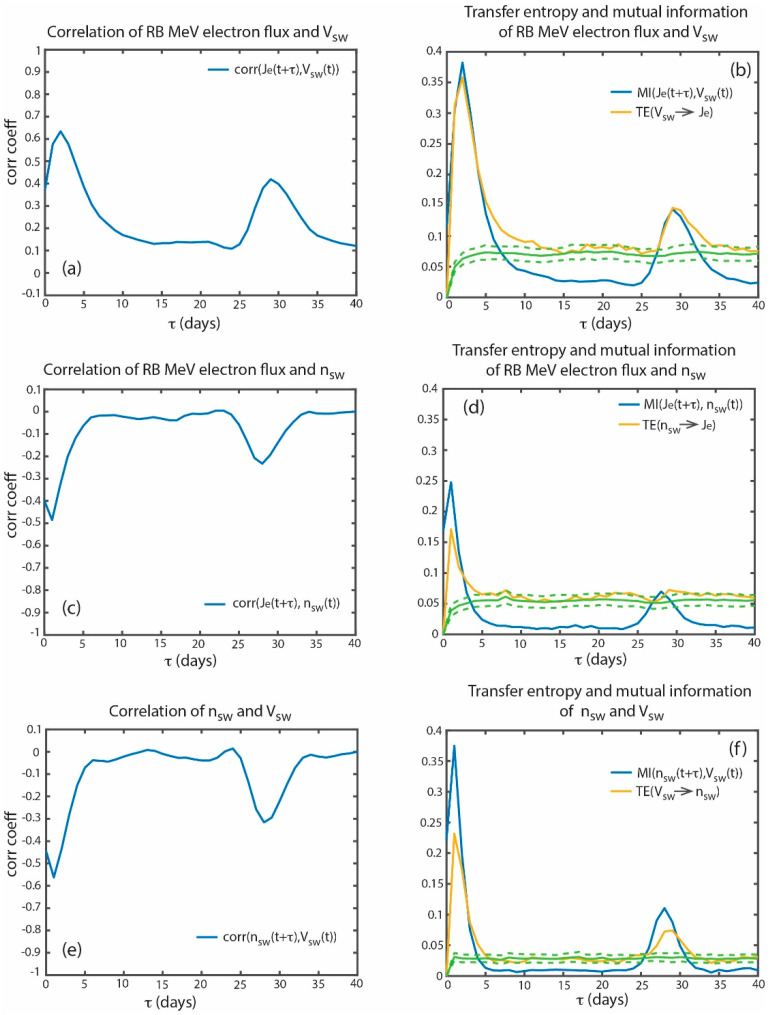
(**a**) Correlation coefficient of [*J_e_*(*t* + *τ*), *V_sw_*(*t*)]. (**b**) MI[*J_e_*(*t* + *τ*), *V_sw_*(*t*)] (blue) and TE[*J_e_*(*t* + *τ*), *V_sw_*(*t*)] (yellow). The transfer of information from *V_sw_* to *J_e_* [TE (*V_sw_* → *J_e_*)] peaks at *τ_max_* = 2 days. (**c**) Correlation coefficient of [*J_e_*(*t* + *τ*), *n_sw_*(*t*)]. (**d**) MI[*J_e_*(*t* + *τ*), *n_sw_*(*t*)] (blue) and TE[*J_e_*(*t* + *τ*), *n_sw_*(*t*)] (yellow). The transfer of information from *n_sw_* to *J_e_* [TE (*n_sw_* → *J_e_*)] peaks at *τ_max_* = 1 day. (**e**) Correlation coefficient of [*n_sw_*(*t* + *τ*), *V_sw_*(*t*)]. (**f**) MI[*n_sw_*(*t* + *τ*), *V_sw_*(*t*)] (blue) and TE[*n_sw_*(*t* + *τ*), *V_sw_*(*t*)] (yellow). The solid and dashed green curves are the mean and 3σ from the mean of the noise. The transfer of information from *V_sw_* to *n_sw_* [TE (*V_sw_* → *n_sw_*)] peaks at *τ_max_* = 1 day. (adapted from [23]).

In order to see the best response lag time of the radiation belt *J_e_* to *V_sw_*, Figure 2a shows the plot of the corr(*J_e_*(*t* + *τ*), *V_sw_*(*t*)). Note that herein, unless otherwise stated, all linear and nonlinear analyses performed with *J_e_* uses log *J_e_* values. The figure shows that the correlation coefficient peaks at *τ* = 2 day with *r* = 0.63. There is a smaller peak at *τ* = 29 days (*r* = 0.42), which can be attributed to the 27-day synodic solar rotation. Because the large number of data points (*n* > 5772), the two peak correlation coefficients are highly significant with *p* < 0.01 (the probability of two random variables giving a correlation coefficient as large as *r* is <0.01). However, the linear correlational analysis may be inadequate because the solar wind–radiation belt system is nonlinear (Figure 1). Hence, mutual information and transfer entropy need to be calculated. Figure 2b shows MI(*J_e_*(t + τ), *V_sw_*(t)) and TE(*V_sw_* → *J_e_*). The figure shows that information transfer from *V_sw_* to *J_e_* peaks at τ = 2 days. Although in this case, the linear correlation, MI, and TE peak at the same τ, in general this is not always the case (as shown in the solar cycle analysis in Section 5).

In order to get a measure of the significance of TE(*V_sw_* → *J_e_*), we calculate noise = TE[sur(*V_sw_*) → *J_e_*] where sur(*V_sw_*) is the surrogate data of *V_sw_*, which is obtained by randomly permuting the order of the time series array *V_sw_*. The mean and standard deviation of the noise are calculated from an ensemble of 100 random permutations of TE[sur(*V_sw_*) → *J_e_*]. The mean noise and 3σ (standard deviation) from the mean noise are plotted with solid and dashed green curves, respectively, in Figure 2b. TE(*V_sw_* → *J_e_*) peaks at *τ_max_* = 2 days where the peak information transfer (*it_max_*) = 0.30, signal to noise ratio (*snr*) = 5.7 and significance = 94σ where *it_max_* = peak–mean noise, *snr* = peak/mean noise and significance = *it_max_*/σ(noise). From the *snr*, *it_max_*, and significance, we conclude that there is a significant transfer of information from *V_sw_* to *J_e_* with a 2-day delay. Note that the linear correlation, MI, and TE analyses are consistent with the previous studies [55,56,57,58,59,60,61].

In contrast to *V_sw_*, which correlates with *J_e_*, solar wind density (*n_sw_*) anticorrelates with *J_e_* for reasons that are not entirely clear [49,61]. An increase in *n_sw_* would increase solar wind dynamic pressure (*P_dyn_*), which, in turn, would push the magnetopause inward, leading to electron losses at the high *L* shell [56]. Furthermore, the magnetopause compression would drive ULF waves [62,63,64] leading to fast radial diffusion, which redistributes the losses to lower *L* shells, including at geosynchronous orbit [49,65,66]. *Ukhorskiy* et al. [67] used a test particle simulation to demonstrate this scenario, which is known as magnetopause shadowing.

Figure 2c shows that the anticorrelation between *n_sw_* and *J_e_* minimizes at *τ* = 1 day (r = −0.49). Figure 2d shows that MI(*J_e_*, *n_sw_*) and TE(*n_sw_* → *J_e_*) peak also at τ = 1 day (*it_max_* = 0.13, *snr* = 4.4 and significance = 42σ). This result is consistent with [60], which finds that *J_e_* has the strongest dependence on *n_sw_* with a lag of 1 day.

However, *V_sw_* and *n_sw_* anti-correlate with each other [68]. Figure 2e shows that the anticorrelation between *V_sw_* and *n_sw_* minimizes at *τ* = 1 day (r = −0.56). Figure 2f shows that MI(*n_sw_*(*t* + *τ*), *V_sw_*(*t*)) and TE(*V_sw_* → *n_sw_*) peak also at *τ* = 1 day (*it_max_* = 0.20, *snr* = 7.4 and significance = 95σ). There is a smaller peak at *τ* = 28 days, which can be attributed to the 27 day synodic solar rotation.

The anticorrelation of *V_sw_* and *n_sw_* with a lag of 1 day complicates the interpretation of the driving of the *J_e_*. If *V_sw_* is causally correlated with *J_e_* with a lag of 2 days, the anticorrelation between *J_e_* and *n_sw_* with a lag of 1 day could simply just be coincidence. Conversely, if *n_sw_* is causally anticorrelated with *J_e_* with a lag of 1 day, the correlation between *V_sw_* and *J_e_* could simply just be coincidence. On the other hand, from Figure 2, we cannot rule out the possibility that both *V_sw_* and *n_sw_* can exert influence on the *J_e_*. If that is the case, how can we untangle the effects of *V_sw_* from *n_sw_* and which of these two parameters exert more influence on *J_e_*?

### 4.1. Untangling the Drivers of the Radiation Belt J_e_

**Figure 3:**
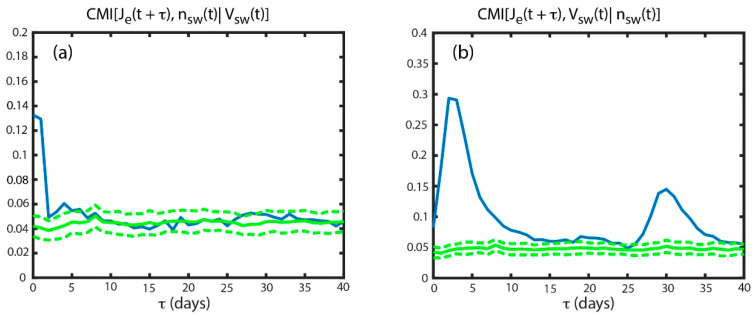
Blue curve showing (**a**) CMI[*J_e_*(*t* + τ), *n_sw_*(*t*) | *V_sw_*(*t*)], and (**b**) CMI[*J_e_*(*t* + *τ*), *V_sw_*(*t*) | *n_sw_*(*t*)]. The solid and dashed green curves are the mean and 3σ from the mean of the noise. (**a**) Unlike MI(*J_e_*, *n_sw_*), which peaks at *τ_max_* = 1 day, CMI[*J_e_*(*t* + *τ*), *n_sw_*(*t*) | *V_sw_*(*t*)] peaks at *τ_max_* = 0 day (*it_max_* = 0.091). The smaller *τ_max_* occurs because CMI removes the effect of *V_sw_* on *J_e_* (see text). (**b**) The peak in CMI[*J_e_*(*t* + *τ*), *V_sw_*(*t*) | *n_sw_*(*t*)] (*it_max_* = 0.25) is broader than that of MI(*J_e_*, *V_sw_*) in Figure 2b because CMI removes the effect of *n_sw_*, which anticorrelates with *J_e_*. *V_sw_* transfers about 2.7 times more information to *J_e_* than *n_sw_*. (from [23]).

In an attempt to address the above questions, we use conditional mutual information, CMI, to untangle the effects of *V_sw_* from *n_sw_* and vice versa. To calculate how much information flows from *n_sw_* to *J_e_*, given *V_sw_*, we calculate CMI[*J_e_*(*t* + *τ*), *n_sw_*(*t*)|*V_sw_*(*t*)], which is plotted as blue curve in Figure 3a. Using a similar approach as for TE, we determine the noise level of the surrogates: CMI[*J_e_*(*t* + *τ*), sur[*n_sw_*(*t*)] | *V_sw_*(*t*)]. The mean and σ of the noise are calculated in the same manner as TE and used to determine the significance of the results. The mean noise and 3σ are plotted as solid and dashed green curves respectively. Figure 3a shows that CMI[*J_e_*(*t* + *τ*), *n_sw_*(*t*) | *V_sw_*(*t*)] peaks at *τ_max_* = 0 day with *it_max_* = 0.091 and *snr* = 3.2. The *τ_max_* = 0 day suggests that *J_e_* response lag time to *n_sw_* is less than 24 h. However, Figure 3a shows that the peak is rather broad, suggesting that the *J_e_* response is still significant at *τ* = 1 day.

Earlier, we establish that *J_e_*(*t* + 2 days) correlates with *V_sw_*(*t*), *J_e_*(*t* + 1 day) anticorrelates with *n_sw_*(*t*), but *n_sw_*(*t* + 1 day) anticorrelates with *V_sw_*(*t*) (Figure 2). In Figure 2d, some of the information in the MI(*J_e_*(*t* + *τ*), *n_sw_*(*t*)) at *τ* = 1–2 days actually come from *V_sw_*. MI(*J_e_*(*t* + *τ*), *V_sw_*(*t*)) peaks at *τ* = 2–3 days. Because *V_sw_* anticorrelates with *n_sw_* at *τ* = 1 day, the information in MI(*J_e_*(*t* + *τ*), *V_sw_*(*t*)) at *τ* = 2–3 days would appear in MI(*J_e_*(*t* + *τ*), *n_sw_*(*t*)) at *τ* = 1–2 days. Subtracting this information from MI(*J_e_*(*t* + *τ*), *n_sw_*(*t*)) at *τ* = 1–2 days would allow the MI(*J_e_*(*t* + *τ*), *n_sw_*(*t*)) at *τ* = 0 to become the tallest peak, as shown in Figure 3a. MI(*J_e_*(*t* + *τ*), *n_sw_*(*t*)) has *it_max_* = 0.21, but removing the effects of *V_sw_*, the *it_max_* drops ~57% to 0.091 (*it_max_* of (CMI[*J_e_*(*t* + 0 day), *n_sw_*(*t*)| *V_sw_*(*t*)] is 0.091).

Conversely, some of the effects attributed to *V_sw_* can be attributed to *n_sw_*, but the effect of *n_sw_* is smaller. To see this, we calculate CMI[*J_e_*(*t* + τ), *V_sw_*(*t*)|*n_sw_*(*t*)], which is plotted in Figure 3b as solid blue curve. The blue curve peaks at *τ* = 2 days with *it_max_* = 0.25 which is about 2.7 times larger than the *it_max_* of 0.091 for CMI[*J_e_*(*t* + *τ*), *n_sw_*(*t*)| *V_sw_*(*t*)]. Thus, *V_sw_* transfers more information to *J_e_* than *n_sw_* does. MI(*J_e_*(*t* + *τ*), *V_sw_*(*t*)) has *it_max_* = 0.32, but removing the effects of *n_sw_*, the *it_max_* drops only ~22% to 0.25 (*it_max_* of CMI[*J_e_*(*t* + 2 days), *V_sw_*(*t*)| *n_sw_*(*t*)] = 0.25).

The reason for the broader peak in Figure 3b is that there is a significant information transfer from *n_sw_* to *J_e_* at *τ* = 0–1 day, but it falls off rapidly at larger τ. Because the anticorrelation between *V_sw_* and *n_sw_* has a one day lag, removing the effects of *n_sw_* would lower MI(*J_e_*(*t* + *τ*), *V_sw_*(*t*)) at τ = 1–2 days. So, *it* for MI(*J_e_*(*t* + *τ*), *V_sw_*(*t*)) at *τ* = 1, 2, and 3 are 0.25, 0.32, and 0.23, respectively, whereas the corresponding values for CMI[*J_e_*(*t* + τ), *V_sw_*(*t*)| *n_sw_*(*t*)] are 0.14, 0.25, and 0.24, respectively. Note that, at *τ* = 1 and 2 there are reductions in information while at *τ* = 3, the information is more or less the same (the difference is within one σ [~0.01]), leading to a broader peak in the CMI[*J_e_*(*t* + *τ*), *V_sw_*(*t*)| *n_sw_*(*t*)] curve in Figure 3b than that in the MI(*J_e_*, *V_sw_*) curve in Figure 2d.

The above analysis suggests that *V_sw_* is the major driver of *J_e_* and shows the *J_e_* response time lag to *V_sw_* and *n_sw_*. The process to accelerate the electrons to MeV energy range takes 2–3 days, as previously suggested [7,49]. Moreover, based on information transfer from *n_sw_* to *J_e_*, any mechanism for *n_sw_* anticorrelation with *J_e_* has to operate or start operating within 24 hr.

Next, we investigate whether other solar wind parameters also contribute to *J_e_*. We calculate the information transfer from southward Interplanetary Magnetic Field (IMF) *B_z_*, northward IMF *B_z_*, IMF *B_y_*, IMF *B_x_*, |IMF B|, *P_dyn_*, σ(IMF *B*), and solar wind electric field (*E_sw_*) to *J_e_*, given *V_sw_*. The northward (southward) IMF Bz is calculated from the daily average of the hourly IMF *B_z_* when IMF *B_z_* > 0 (IMF *B_z_* < 0). The results are tabulated in Table 1, which ranks various solar wind parameters based on the *it_max_*. Thus, the ranking gives the importance of each solar wind parameter based on the information transfer to *J_e_*. Table 1 also lists *τ_max_* which signifies the lag time when information transfer to *J_e_* maximizes.

Note that the ranking in Table 1 is obtained with daily resolution data. It is possible that the ranking of some parameters may change if the data are analyzed at higher time resolution. For example, some studies showed that southward IMF *B_z_* can influence *J_e_* [69,70,71] but southward IMF *B_z_* is only ranked number 5 in Table 1. IMF fluctuates with periods of northward and southward IMF at minutes or tens of minutes timescale. Thus, the low ranking of the southward IMF *B_z_* most likely result from the fluctuations of IMF *B_z_* within one day period [7,56,60]. Consistent with our result, *Li* et al. [56] found IMF *B_z_* is poorly correlated with *J_e_* at daily resolution.

In Figure 3b, the CMI[*J_e_*(*t* + *τ*), *V_sw_*(*t*)| *n_sw_*(*t*)] curve shows that *V_sw_* has little influence on the geosynchronous MeV electrons after a delay of 7–10 days. Thus, using *V_sw_*, the prediction or information horizon for *J_e_* is about 7–10 days. Figure 3a shows that using *n_sw_*, the prediction horizon for *J_e_* is about 2 days.

In applying our information theoretical tools, the number of bins (*n_b_*) need to be chosen appropriately. *Sturges* [72] proposes that for a normal distribution, optimal *n_b_* = log_2_(*n*) + 1 and bin width (*w*) = range/*n_b_*, where *n* = number of points in the dataset, range = maximum value–minimum value of the points. In practice, there is usually a range of *n_b_* that would work. Using *Sturges* [72] formula, with roughly 6400 points, *n_b_* ~13.6. For the present study, we find that *n_b_* = 10 to 15 would work well. Having too few bins would lump too many points into the same bin, leading to loss of information. Conversely, having too many bins would leave many bins with 0 or a low number of points, which also leads to loss of information. For the present study, we choose *n_b_* = 10.

### 4.2. The Triangle Distribution

*Reeves* et al. [7] was the first to note the right triangle distribution exhibited in Figure 1 panels a–c (the contours in Figure 1d qualitatively look more like parallelograms than right triangles). The left-hand side of the triangle forms because *V_sw_* rarely goes below 300 km s^−1^. The hypotenuse of the triangle suggests that the lower limit of *J_e_* more or less increases with *V_sw_*. The top side of the triangle suggests that *J_e_* saturates, which can be attributed to local instabilities [73]. As noted by *Reeves* et al. [7], the most interesting and perhaps mystifying aspect of the triangle distribution is that high *J_e_* is observed for all *V_sw_* conditions and the variability of *J_e_* at lower *V_sw_* is much larger than that at higher *V_sw_*.

**Figure 4:**
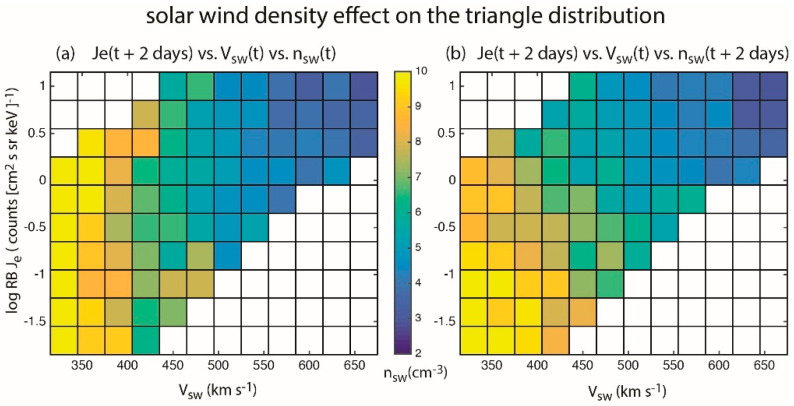
Points in *J_e_*(*t* + 2 days) vs. *V_sw_*(*t*) distribution in Figure 1c are binned in 0.3 counts (cm^2^ s sr keV)^−1^ × 30 km s^−1^ bins. Each point is assigned its *n_sw_*(*t*) and *n_sw_*(*t* + 2 days) values. The latter has no time shift with respect to *J_e_* such that information transfer from *n_sw_* to *J_e_* maximizes. (**a**) shows the mean *n_sw_*(*t*) while (**b**) shows the mean *n_sw_*(*t* + 2 days) of each bin. In (**a**), the density gradient is mainly in the *x* direction due to the anticorrelation between *n_sw_* and *V_sw_*. However, in (**b**), there are density gradients in *x* and *y* direction. The latter can be attributed to *P_dyn_* and magnetopause shadowing. (from [23].)

We investigate the effect of *n_sw_* on the triangle distribution. We obtain *n_sw_*(*t*) for each point in the log *J_e_*(*t* + 2 days) vs. *V_sw_*(*t*) scatter plot in Figure 1c. The *n_sw_* in the figure are then binned in 0.3 counts (cm^2^ s se keV)^−1^ × 30 km s^−1^ bins. Figure 4a shows the mean *n_sw_* of each bin. Bins with fewer than 15 points are not displayed. The most prominent trend in Figure 4a is a strong density gradient in the *x* direction because *n_sw_* anticorrelates with *V_sw_*.

However, our analysis and Figure 3a suggest that the maximum transfer of information from *n_sw_*(*t*) to *J_e_*(*t* + *τ*) occurs at *τ* = 0 day (<24 hr). Hence, instead of assigning *n_sw_*(*t*) to each point in the *J_e_*(*t* + 2 days) vs. *V_sw_*(*t*) plot, we assign *n_sw_*(*t* + 2 days) so that *J_e_* is not time shifted with respect to *n_sw_*. We repeat the same procedure done for Figure 4a and the result is shown in Figure 4b. Now, there are density gradients in both *x* and *y* directions. As in Figure 4a, the density gradient in the *x* direction is due to the anticorrelation of *n_sw_* with *V_sw_*. Figure 4b clearly shows that for *V_sw_* < 500 km s^−1^, larger *n_sw_* hence larger *P_dyn_* can be associated with lower *J_e_* and vice versa. This density gradient in the *y* direction may be attributed to the magnetopause shadowing effect, which rapidly depletes radiation belt fluxes when solar wind pressure is increases. Thus, the triangle distribution can be explained by the correlation of *V_sw_*(*t*) and *J_e_*(*t* + 2 days) and the anticorrelation of *n_sw_*(*t*) and *J_e_*(*t* + 0 day).

## 5. The Solar Cycle

The surface of the Sun has dark regions or spots, commonly called sunspots. The number of sunspots vary cyclically with a period of 11 years, known as solar cycle. Although the cyclical nature of sunspots has been known for over 150 years [74], it is not clear what causes the solar cycle. Predicting *SSN* remains a challenge [75].

**Figure 5:**
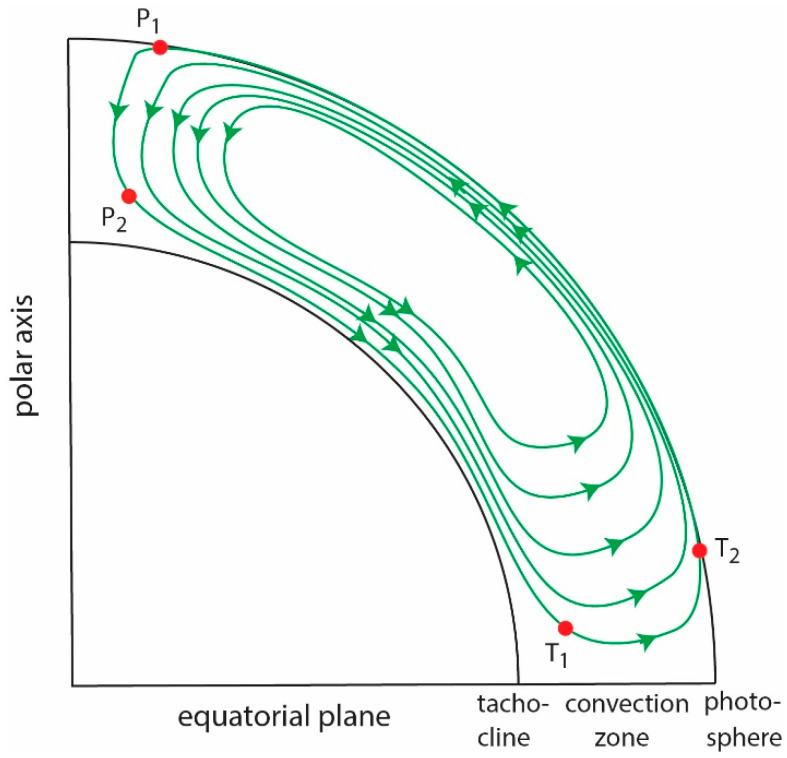
Babcock–Leighton type solar cycle dynamo model. The diagram shows a meridional slice of the sun. The meridional flow is plotted in green with arrows indicating the flow direction. Poloidal field at P1 is advected down to P2 in the convective zone by the meridional flow. The meridional flow advects the field from P2 to T1, while the differential rotation shears the field, converting it to toroidal field. The buoyancy force lifts the toroidal field from T1 to the photosphere at T2, producing sunspots. The sunspots decay into poloidal field, which is carried by the meridional flow to the T1 and the cycle starts over again. (from [25]).

Most solar cycle models today are based on the Babcock–Leighton model [76,77,78]. This is depicted in a diagram in Figure 5 for one hemisphere (see review in [79]). Near the solar minimum, the magnetic field, which has a poloidal configuration, is advected downward to the base of the convection zone, possibly as far down as the tachocline, by the meridional flow from P1 to P2 in Figure 5. From P2, the poloidal field carried by the meridional flow to low latitude (T1). As it does, the latitudinal differential rotation shears the magnetic field, which twists and wraps the field line around the sun azimuthally, leading to the growth of the toroidal field at low latitude at T1. The twisting and turning motions create regions of strong magnetic field, which choke off the plasma and energy flow into the regions and which become buoyant. The buoyancy force pushes these strong magnetic field regions to the surface (photosphere), where they appear at T2. The Coriolis force acting on the rising toroidal field causes the emerging fluxes to appear as pairs of sunspots with a slight tilt such that the following spot of the pair tends to be further from the equator than the preceding spot (Joy’s law), leading to the growth of the poloidal field. As the sunspots decay, the equatorward spot moves equatorward and the poleward spot moves poleward. The equatorward field cancels the one with the opposite polarity from the opposite hemisphere. The meridional flow carries the poleward poloidal field, which has the opposite polarity than that at P1, and which destroys some of the old poloidal fields at P1. This process continues until a new poloidal field with the opposite polarity is established, which commences a new solar cycle. An example of a mathematical formulation of Babcock–Leighton type model can be seen in a flux transport dynamo model [80].

From the description above, it is clear that the meridional flow, which acts as a conveyor belt that circulates the magnetic fluxes, plays an important role in the solar cycle [81,82,83]. Another important parameter is the polar field. Some flux transport dynamo models input the strength of the polar field at solar minimum, which in turn, determines the amplitude of the next solar cycle [82,84,85]. The flux emergence, which can be proxied by *SSN*, is yet another important parameter as it can affect and be affected by the polar field and meridional flow [82].

**Figure 6:**
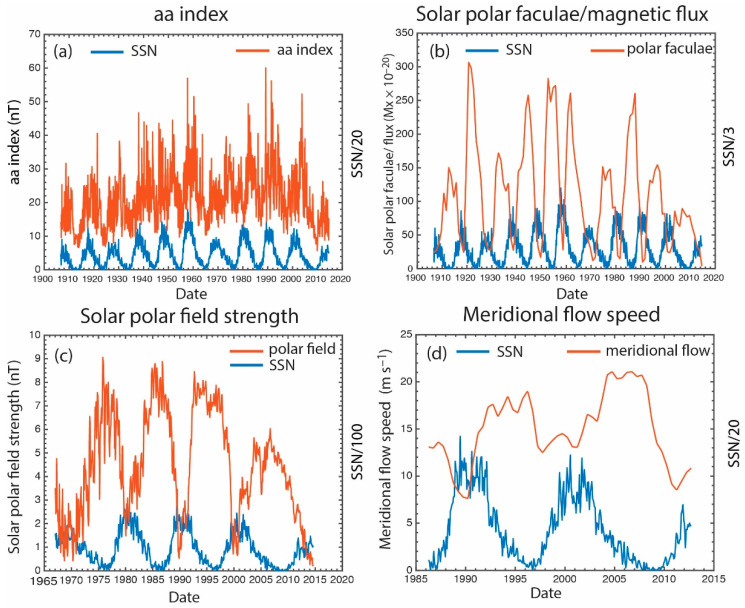
Solar cycle variations of (**a**) *aa* index; (**b**) the solar polar faculae calibrated to SOHO MDI polar magnetic flux [9]; (**c**) the solar polar field strength; (**d**) the meridional flow. These parameters are plotted in red curves whereas the *SSN* is plotted in the blue curves. The *SSN* has been scaled by a different factor in each figure as indicated by the right *y*-axis label in order to enhance viewing. (adapted from [25]).

The information transfer from the polar field, *aa* index, polar faculae, and meridional flow to *SSN* and vice versa are investigated. These parameters are plotted in Figure 6. Because the meridional flow circulates the magnetic fluxes continuously, these parameters, which are magnetic in nature, can affect each other bidirectionally, which poses a challenge. That is, a parameter *x* can affect *y*, but *y* can also affect *x* some time later. This situation is similar to the well-known predator–prey dynamics [86], where each population affects the other in a causal manner. However, in a situation with multiple species the causal relationship between any two species may have a directionality if one species has a stronger effect on the other species. These bidirectionalities need to be untangled or taken into account with transfer entropy [4].

Our methodology does not a priori assume the relationships between any of the parameters. For example, rather than performing superposed epoch analysis with solar minimum as t = 0, which assumes implicitly or explicitly that solar minimum is the start of the cycle, as done in some studies, we treat the data simply as long time series. In our approach, the solar cycle is a sequence of events (which has occurred for centuries or at least since the data became available) where the meridional flow acting like a perpetual conveyor belt that circulates the magnetic fluxes continuously from the polar region in the photosphere, to low latitude in the convection zone, to the low latitude photosphere, and finally back to the polar region (Figure 5). Therefore, there is no beginning or end to the solar cycle and our results can be viewed as pertaining to the average dynamics of the Sun during the entire period for which the data are analyzed. We are cognizant that the behavior of an individual cycle may vary from one cycle to the next. Hence, care needs to be exercised when using our results to interpret the dynamics of a particular solar cycle. Our approach naturally lends itself to the investigations of the long-term effects (more than a few or several cycles) of one parameter on another, as done in Section 5.4.

### 5.1. aa Index and SSN

In the study of the Earth’s magnetosphere, it has been known for decades that the amount of active regions at the Sun, proxied by *SSN*, can cause variations in geomagnetic activity, e.g., the *aa* index. For example, Coronal Mass Ejections (CMEs) and solar wind high speed streams that occur more frequently during solar maximum and in the declining phase of solar maximum, respectively, can increase the *aa* index. However, it was suggested that the low level geomagnetic activity is correlated with the strength of interplanetary magnetic field (IMF), which is related to the solar polar field [87,88,89]. In turn, the polar field can affect the sunspot productions sometime later. *Ohl* [90] and *Hathaway* et al. [91] showed that the *aa* index at solar minima is a good predictor of the maximum *SSN* of the next cycle. *Wang and Sheeley* [92] reported that when averaged over timescales longer than a month, the *aa* index is highly correlated with the radial IMF (B_r_), which is proportional to the Sun’s total open flux, which in turn, is correlated with the Sun’s dipole field strength. For these reasons, the *aa* index may contain (albeit indirect) some information about the polar field, which facilitates information flow from the *aa* index to *SSN*. This scenario is investigated with information theory.

**Figure 7:**
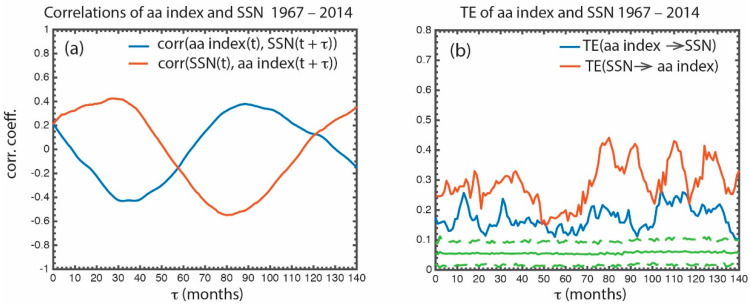
(**a**) Time shifted correlation corr[*aa* index(t), *SSN*(t + τ)] is plotted in blue and corr[*SSN*(t), *aa* index(t + τ)] is plotted in red. The peak |corr[*aa* index(t), *SSN*(t + τ)]| is roughly the same as the peak |corr[*SSN*(t), *aa* index(t + τ)]|. (**b**) TE(*aa* index → *SSN*) is plotted in blue and TE(*SSN* → *aa* index) is plotted in red. TE(*SSN* → *aa* index) > TE(*aa* index → *SSN*), suggesting that more information is transferred from the *SSN* to *aa* index than the other way around. Such information cannot be discerned from the correlations shown in (**a**). The solid and dashed green curves show the mean and 3σ of the noise (see text). The data are for the period 1967–2014. (from [25]).

The time shifted correlation between the *aa* index and *SSN* is calculated. corr[*aa*(t), *SSN*(t + τ] (blue curve) and corr[*SSN*(t), *aa*(t + τ)] (red curve) are plotted in Figure 7a. The red curve shows that *aa*(t + τ) is correlated with *SSN*(t) with a peak correlation at τ ~30–40 months [correlation coefficient (r) = 0.43] and is anticorrelated with *SSN*(t) a half solar cycle later. The peak |r| in both the blue and red curves are significant (*p* < 0.01). Because both the *SSN* and *aa* curves are cyclical and attain approximately the same maximum |r| in Figure 7a, it is virtually impossible to discern from the figure whether the *aa* index is causally related to the *SSN* or the other way around.

However, the transfer entropy conveys a totally different picture. The transfer entropy from *aa* to *SSN* [TE(*aa* → *SSN*)] (blue curve) and from *SSN* to *aa* [TE(*SSN* → *aa*)] (red curve) is presented in Figure 7b. The figure shows that indeed there is a bidirectional flow of information from the *aa* index to *SSN* and vice versa. As would be expected, Figure 7b shows that there is more information transfer from the *SSN* to *aa* index than the other way around, which the standard correlational analysis cannot establish (Figure 7a). Although the transfer of information from the *SSN* to *aa* index has a peak at τ ~ 30–40 months, it has larger peaks at τ > 70 months.

The *aa* index carries information about the Earth’s magnetospheric dynamics, e.g., [12,21,22]. However, the *aa* index also carries some information about its drivers such as the IMF, which is related to the polar field. However, as the blue curve in Figure 7b suggests, only a small amount of information about the polar field is embedded within the *aa* index. The *aa* index would not be a good proxy for the polar field.

TE(*aa* → *SSN*) has peak information transfer (*it_max_*) = 0.20, signal to noise ratio (*snr*) = 4.3, and significance = 14σ. The bidirectional flow of information between two solar cycle parameters is typical in solar parameters discussed herein. The *aa* index–*SSN* example presented here illustrates the power of our methodology.

### 5.2. Polar Field and SSN

**Figure 8:**
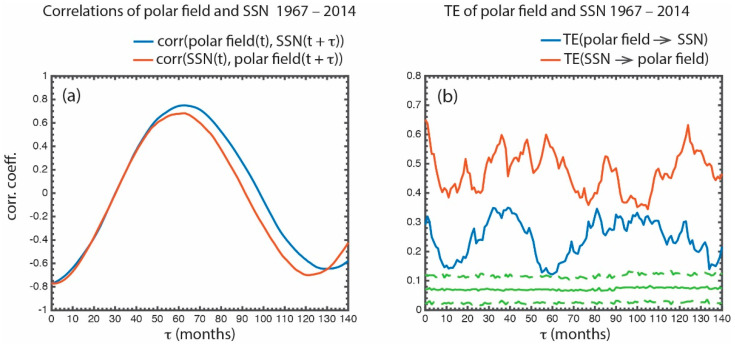
(**a**) Time shifted correlation corr[polar field(t), *SSN*(t + τ)] is plotted in blue and corr[*SSN*(t), polar field(t + τ)] is plotted in red. They both reach minima at τ ~0 month and maxima at τ ~60–70 months (half solar cycle period) because the polar field and *SSN* tend to be 180° out of phase with each other. (**b**) TE(polar field → *SSN*) is plotted in blue and TE(*SSN* → polar field) is plotted in red. The format is the same as in Figure 7. The transfer of information from the polar field to *SSN* peaks at τ ~30–40 months. There is significant information transfer from the *SSN* to polar field as well. The solid and dashed green curves show the mean and 3σ of the noise. The data are for the period 1967–2014. (from [25]).

The same analysis done in Section 5.1 is repeated with the polar field and *SSN* data 1967–2014. corr[polar field(t), *SSN*(t + τ) (blue curve) and corr[*SSN*(t), polar field(t + τ)] (red curve) are presented in Figure 8a. The correlation coefficients for both curves peak at τ ~60–70 months (half solar cycle period). This result may be consistent with the Babcock–Leighton model in which the polar field at solar minimum determines the *SSN* at the next maximum. However, Figure 8b shows that TE(polar field → *SSN*) peaks at τ ~30–40 months (blue curve). Note that TE(polar field → *SSN*) calculates the information transfer from the polar field to *SSN*, given that the present and past values of the *SSN* are known, which differs from the linear correlation (Figure 8a). The lag time of ~30–40 months is interesting because some models have assumed a lag time of half solar cycle period (66 months). The secondary peak at τ ~0–2 months results from the anticorrelation of the polar field with the *SSN* with little or no lag.

The lag times of 30–40 months seen in the blue curve in Figure 8b may be related to the time that it takes for the polar field to advect downward and equatorward in the convection zone and to reemerge at the low latitude photosphere. In some models, this time is assumed to be a half the solar cycle period, but our result shows that it can be shorter. This result suggests that perhaps some of the fluxes may emerge at a higher latitude in the equatorial region or some fluxes may submerge at a lower latitude in the polar region. The shorter lag may be consistent with the observations that sunspots of the new cycle appear years before the solar maximum [93,94] and flux emergence at high latitudes [95]. Further investigation is needed.

Figure 8b shows that there is also a significant information transfer from the *SSN* to the polar field. In fact, there is more information transfer from the *SSN* to polar field than the other way around. The information transfer from the *SSN* to polar field has a broad peak at τ ~30–60 months and two narrower peaks at τ ~80–90 months and at τ ~120–130 months. The latter (peak at τ ~120–130 months) may be due to the correlation at the ~11-year solar cycle. *Upton and Hathaway* [82] suggested that the amount of flux emergence (proxied by the *SSN*) determines the polar field strength at the next solar minimum. This may be consistent with our result (red curve), but our result shows the maximum response lag times for this process.

The multiple peaks in the red curve in Figure 8b could result from the episodic nature of flux transport to the poles. It is well known that flux is transported to the poles in ‘surge’ with their number and intensity determined by the meridional flow speed [81]. The flux surges take about 2–3 years to reach the poles and seem to last for about 5 years in the period examined, 1967–2014.

All the peaks in the blue and red curves are well above the 3σ from the mean noise (the dashed green line). TE(*polar field* → *SSN*) has peak information transfer (*it_max_*) = 0.28, signal to noise ratio (*snr*) = 5.0, and significance = 19σ.

### 5.3. The Parameters That Control the Polar Field

It is not clear which parameters play the most dominant role in controlling the polar field. In some surface flux transport models [81,92,96,97,98] a larger meridional flow would lead to weaker polar field and a long cycle whereas in some flux transport dynamo models, it would lead to stronger polar field and a short cycle [84,99]. However, recent studies suggested that the polar field in cycle 23/24 may have been determined to a large extent by the flux emergence, e.g., *SSN* [82].

**Figure 9:**
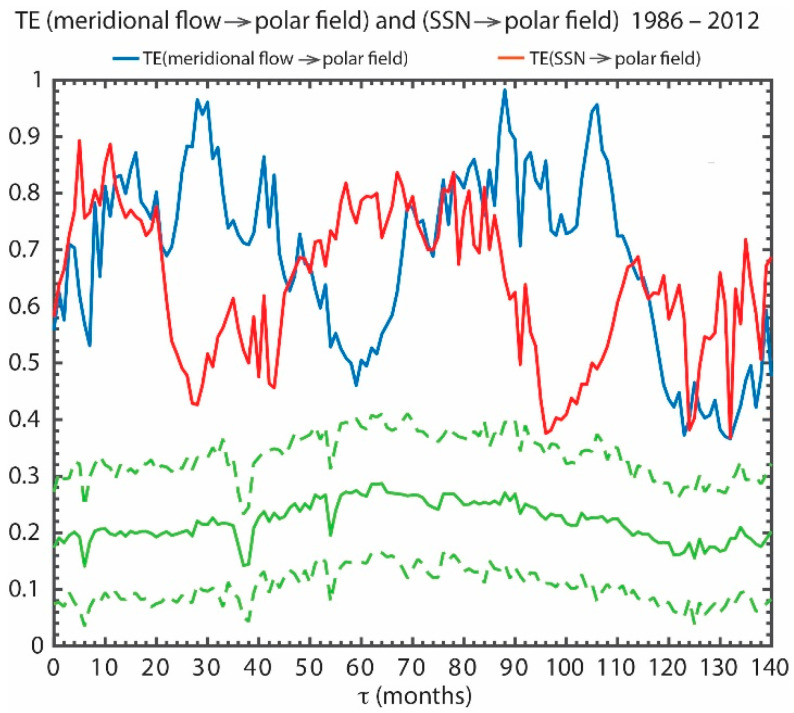
TE(meridional flow → polar field) and TE(*SSN* → polar field) are plotted in blue and red curves, respectively, for the period 1986–2012. The curves are noisy because of the limited availability of the meridional flow data. Both the meridional flow speed and *SSN* (proxy for flux emergence) transfer information to the polar field, but the meridional flow speed transfers more information to the polar field than *SSN* at τ ~28–30 months and τ ~90–110 months. On the other hand, the *SSN* transfers more information to the polar field than the meridional flow at τ ~60–80 months. The solid and dashed green curves show the mean and 3σ of the noise. (from [25]).

Figure 9 presents TE(meridional flow → polar field) (blue curve) and TE(*SSN* → polar field) (red curve). The plot of TE(*SSN* → polar field) for 1967–2014 has already been shown in Figure 8b. However, in order to compare it with the meridional flow speed, it is necessary to analyze the data for the same interval when there are the meridional flow data, namely 1986–2012.

It turns out that there is information flow from both the meridional flow and *SSN* to the polar field, but their relative contribution to the polar field differs at different lag times, as shown in Figure 9. The information transfer from the meridional flow to the polar field is dominant at τ ~28–30 and τ ~90–110 months (almost one solar cycle period) during 1986–2012. The peak at τ ~28–30 months may be consistent with the flux transport dynamo models referenced above and the *Schrijver* et al. surface flux transport model [100]. However, the transfer of information from the *SSN* (proxy for the flux emergence) to the polar field is dominant at τ ~60–80 months (about a half solar cycle period) (smaller τ is obtained for the period 1967–2014), which may be consistent with a newly developed surface flux transport model [82]. Our results provide observational constraints for when the information flow from the meridional flow speed and *SSN* to the polar field should maximize.

All the peaks in the blue and red curves in Figure 9 are well above the 3σ deviation from the mean noise (the dashed green line). For example, peak of TE(meridional flow → polar field) has *it_max_* = 0.71, *snr* = 3.6, and significance = 17σ. The peak of TE(*SSN* → polar field) is lower, but is still significant.

### 5.4. The Importance of the Polar Fields in Last Few Cycles for Predicting SSN

In *Dikpati* et al. solar flux transport dynamo model [101], the polar field is advected down to the base of the convection zone where it remains for 17–21 years instead of just half the solar cycle period as it travels to low latitude. This polar field becomes the seed for toroidal field that emerges in the photosphere as sunspots. Hence, *Dikpati* et al. [101] argued that the polar fields not just from the last cycle, but also from the last 3 cycles should affect the production of sunspots. The importance of the polar fields in the past few cycles is also found in the *Charbonneau and Dikpati* simulation [102], which shows that the toroidal field (proxy for the SSN) at cycle n has the best correlation with the polar field at cycle n–2. This possibility is investigated with information theory. Because the polar field data are available presently for only 4.5 solar cycles, the long-term effect of the polar field is investigated with the polar faculae instead. Studies suggested that polar faculae are as a good proxy for the polar fields [103,104,105,106]. The polar faculae data are available for almost 10 cycles, 1906–2014, which makes it more suitable for the investigation of long-term effects.

**Figure 10:**
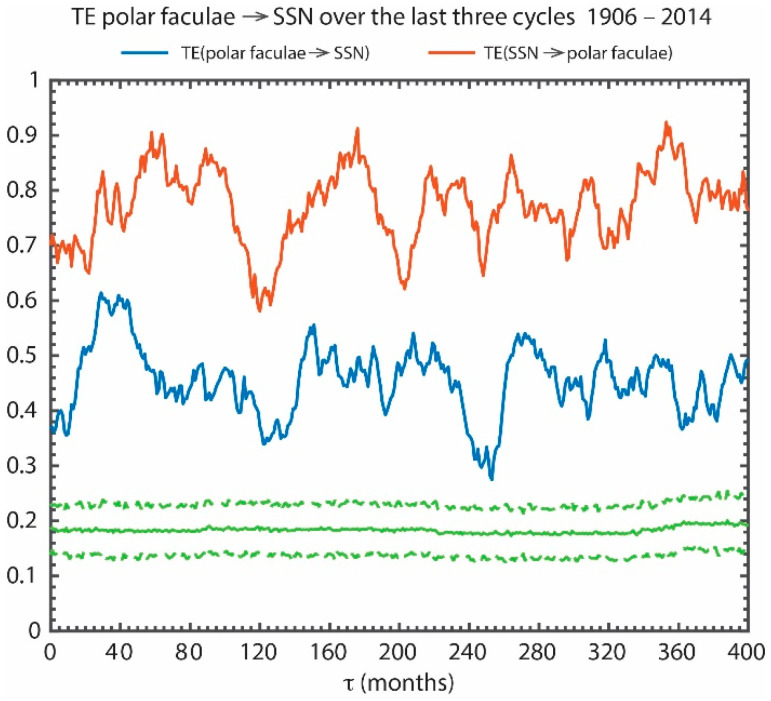
The long-term effect of the polar fields (as proxied by the polar faculae) on sunspot production. TE(polar faculae → *SSN*) and TE(*SSN* → polar faculae) are plotted in blue and red curves, respectively, for the period 1906–2014. The transfer of information from the polar faculae (proxy for the polar fields) to *SSN* peaks at *τ* ~30–40 months, but thereafter it persists for at least 400 months (~3 solar cycle period) albeit at lower level. The solid and dashed green curves show the mean and 3σ of the noise. There is also a long-term effect of the *SSN* on polar faculae. (from [25]).

Figure 10 presents TE(polar faculae → *SSN*) (blue curve) and TE(*SSN* → polar faculae) (red curve). The figure shows that the transfer of information from the polar faculae to *SSN* peaks at about *τ* ~30–40 months, consistent with our analysis using the polar field data with shorter timespans in Section 5.2. Thereafter, the information transfer drops to a lower level, but it persists for at least 400 months, which lends support to the idea that the information about the production of the *SSN* can be found not just from the polar field from the last cycle, but the polar fields from at least the last 3 cycles. However, this may just simply be a reflection of the fact that the solar magnetic field is recycled from one cycle to the next and magnetic flux conservation. Interestingly, TE(polar faculae → *SSN*) has minima at *τ* ~120–140 (roughly one solar cycle period) and at *τ* ~240–260 (roughly two solar cycle periods).

Figure 10 shows that there is actually more information transfer from the *SSN* to polar faculae than from the polar faculae to *SSN*, as found earlier with the polar field data with shorter timespans. The figure shows that the information flow from the *SSN* to polar faculae does peak at around half solar cycle (~50–70 months), but the curve has also other peaks at longer lag times. However, this longer information horizon could perhaps be expected if there is a long-term effect of the polar fields on *SSN* because the meridional flow circulates the fluxes with a period of a solar cycle.

The analysis has been extended to *τ* > 400 months. The result indicates that there is still low level information transfer from the polar faculae to the *SSN* at *τ* > 400 months (>3 solar cycle period), but the TE gets noisier as τ gets larger because the polar faculae data only span for about 10 cycles.

All the peaks in the blue and red curves are well above the 3*σ* from the mean noise (the dashed green line). TE(polar faculae → *SSN*) has *it_max_* = 0.43, *snr* = 3.3, and significance = 28*σ*.

## 6. Concluding Remarks

In the present paper, we show how information theory can be useful in the studies of solar and space physics. Our methodology can generally be applied to a large number of problems because it does not assume a priori the underlying physics of the system. Our findings of information transfer from one parameter to another and the response lag time can provide insights into the physical relationships between the two parameters and provide constraints to models.

We present examples from two applications, one in solar cycle dynamics and one in radiation belt dynamics, each with its own unique challenge. These two examples are intended to show the breadth of the applications of the methodology.

In the radiation belt study, the challenge is that several solar wind drivers can affect the radiation belt flux, *J_e_*, simultaneously. We show how information theory can be useful to untangle the drivers of the solar wind–radiation belt system, identify causal parameters, and the system response lag times to the drivers. One of the conclusions is that the radiation belt electron response lag times to *V_sw_* and *n_sw_* are 2 and 0 day, respectively. We show that the nonlinearity and the high variability of *J_e_* in the triangle distribution can be better understood if we take these response time lags into account.

The solar cycle study presents a particular challenge not found in the radiation belt study, which is that each parameter can affect one another bidirectionally because the solar magnetic field is largely recycled from one cycle to the next. In order to understand the dynamics, these bidirectionalities need to be considered. The simple correlational analysis would not be able to untangle these bidirectionalities, but transfer entropy proves more useful and provides more insights. For example, although the time shifted correlations between aa index and *SSN* peak at roughly the same value in both directions, the transfer entropy analysis presents a different picture. More information is transferred from *SSN* to *aa index* than the other way around, as would be expected because aa index is a poor proxy of the Sun’s polar field.

Moreover, we show that transfer of information from the polar field to *SSN* peaks at 30–40 months. Both, the meridional flow and *SSN* transfer information to the polar field, but each parameter dominates at different time lags. The meridional flow transfers more information to the polar field at τ ~28–30 months and τ ~ 90–110 months, whereas *SSN* transfers more information at τ ~ 60–80 months. Finally, there seems to be support for the idea that the polar field from the last 3 cycles can affect the production of the sunspots because the meridional flow at the bottom of the convective zone is slower than at the top. Our entropy-based approach can be useful to solar cycle modelers and theorists because (1) it can establish which parameters are causally related to the *SSN*, (2) it can rank the parameters based on the information transfer to the *SSN*, and (3) it can provide the *SSN*’s response lag times to these parameters. All of these can provide observational constraints to solar cycle models.

## Figures and Tables

**Table 1:** Ranking of the solar wind parameters based on information transfer to geosynchronous Mev electron flux (*J_e_*) at *τ_max_*, where *τ_max_* is the lag time when the information transfer peaks. Parameters 2–9 are calculated from CMI[*J_e_*(*t* + *τ*), *x*(*t*) | *V_sw_*(*t*)] whereas parameter 1 is calculated from CMI[*J_e_*(*t* + *τ*), *V_sw_*(*t*) | *n_sw_*(*t*)], where *x* = parameter 2–9. The peak information transfer (*it_max_*) = peak – mean noise, the signal to noise ratio = peak/noise, and significance = *it_max_*/σ(noise). Noise is calculated from surrogate data (see Section 4). The prediction horizon gives the lag time when there is no information transfer from the solar wind parameter to *J_e_*. Note that *n_sw_* and *P_dyn_* are both ranked at number 3 because they have similar *it_max_* (the effect of *V_sw_* has been removed). ^*^ excluding the effect of solar rotation. (from [23].)

Rank	Solar Wind Parameters	Peak Information Transfer (*it_max_*)	Signal to Noise Ratio at *τ_max_*	Significance at *τ_max_* (σ)	*τ_max_* (Days)	Prediction Horizon (Days)
1	*V_sw_*	0.25	6.6	94	2	10 ^*^
2	IMF |**B**|	0.12	3.9	48	0	2
3	*P_dyn_*	0.092	3.4	35	0	2
3	*n_sw_*	0.091	3.2	34	0	2
4	*σ*(IMF *B*)	0.075	3.9	48	0	2
5	IMF *B_z_* < 0	0.064	2.7	26	0	2
6	*E_sw_*	0.056	2.9	22	1	5
7	IMF *B_y_*	0.052	2.3	20	0	2
8	IMF *B_z_* > 0	0.048	3.1	22	0	2
9	IMF *B_x_*	0.044	2.2	19	0	2

## Supplementary Materials

Supplementary File 1
