# Supplementary material for: Applications of Information Theory in Solar and Space Physics
Source: Entropy (Basel). 2019 Feb 1;21(2):140. doi: 10.3390/e21020140 (PMC7514618; doi:10.3390/e21020140)
Supplement: Supplementary File 1 [file entropy-21-00140-s001.pdf]

Supplementary

Applications of information theory in solar and space physics

Simon Wing [1,*] and Jay R. Johnson[2]

[1] The Johns Hopkins University, Applied Physics Laboratory, Laurel, Maryland, USA

[2] Andrews University, Berrien Springs, Michigan, USA; jrj@andrews.edu

* Correspondence: simon.wing@jhuapl.edu; Tel.: +xx-xxx-xxx-xxxx

Received: date; Accepted: date; Published: date

Table 1. Ranking of the importance of the solar wind parameters based on information transfer to geosynchronous Mev electron flux (J_e) at τ_{max}, where τ_{max} is the lag time when the information transfer peaks. Parameters 1–9 are calculated from CMI[$J_e(t + \tau)$, $x(t)$ | $V_{sw}(t)$] whereas parameter 1 is calculated from CMI[$J_e(t + \tau)$, $V_{sw}(t)$ | $n_{sw}(t)$], where x = parameter 1–9. The peak information transfer (it_{max}) = peak – mean noise, the signal to noise ratio = peak/noise, and significance = it_{max}/σ(noise). Noise is calculated from surrogate data (see Section 6.4.1). The prediction horizon gives the lag time when there is no information transfer from the solar wind parameter to J_e. Note that n_{sw} and P_{dyn} are both ranked at number 3 because they have similar it_{max} (the effect of V_{sw} has been removed [see Section 6.5.3]). Northward IMF has slightly higher *snr* than southward IMF because northward IMF has lower noise level than southward IMF.

rank	solar wind parameters	peak information transfer (it_{max})	signal to noise ratio at τ_{max}	Significance at τ_{max} (σ)	τ_{max} (days)	prediction horizon (days)
1	V_{sw}	0.25	6.6	94	2	10*
2	IMF |**B**|	0.12	3.9	48	0	2
3	P_{dyn}	0.092	3.4	35	0	2
3	n_{sw}	0.091	3.2	34	0	2
4	σ(IMF B)	0.075	3.9	48	0	2
5	IMF $B_z < 0$	0.064	2.7	26	0	2
6	E_{sw}	0.056	2.9	22	1	5
7	IMF B_y	0.052	2.3	20	0	2
8	IMF $B_z > 0$	0.048	3.1	22	0	2
9	IMF B_x	0.044	2.2	19	0	2

*excluding the effect of solar rotation.

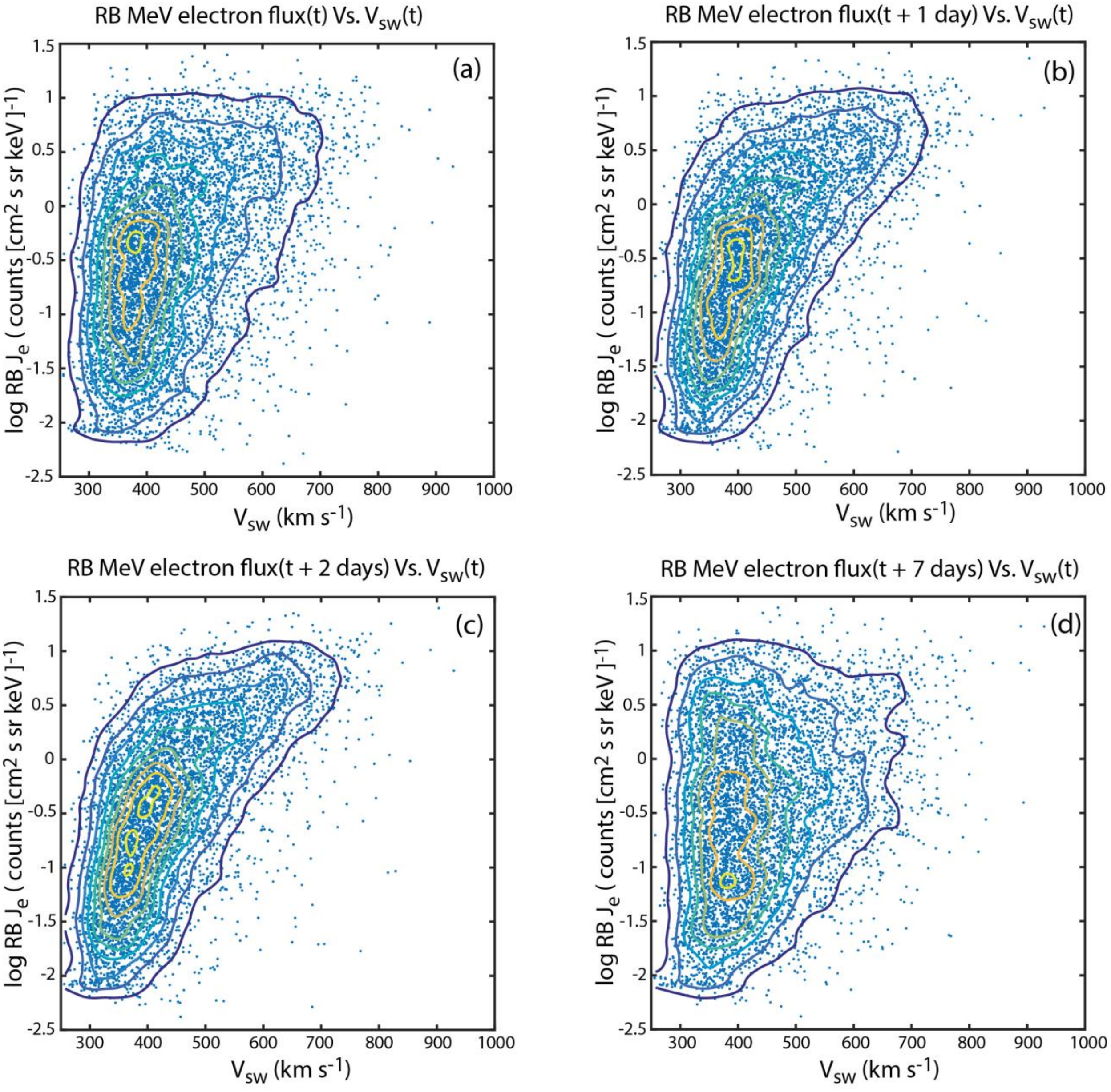

Figure 1. Scatter plots of log $J_e(t + \tau)$ vs. $V_{sw}(t)$ for $\tau = 0$, 1, 2, and 7 days in panels (a), (b), (c), and (d), respectively. The data points are overlain with density contours showing the nonlinear trends. The panels show that J_e has dependence on V_{sw} for $\tau = 0$, 1, and 2 days and the dependence is strongest for $\tau = 2$ days. (d) At large τ, e.g., $\tau = 7$ day, J_e dependence on V_{sw} is very weak. The triangle distribution (*Reeves et al*., 2011) can be seen in panels (a), (b), and (c). This is essentially the same as Figure 9 in *Reeves et al*. (2011), except that no density contours are drawn and Figure 6.1d plots $\tau = 7$ days instead of $\tau = 3$ days. (from Wing et al., 2016.).

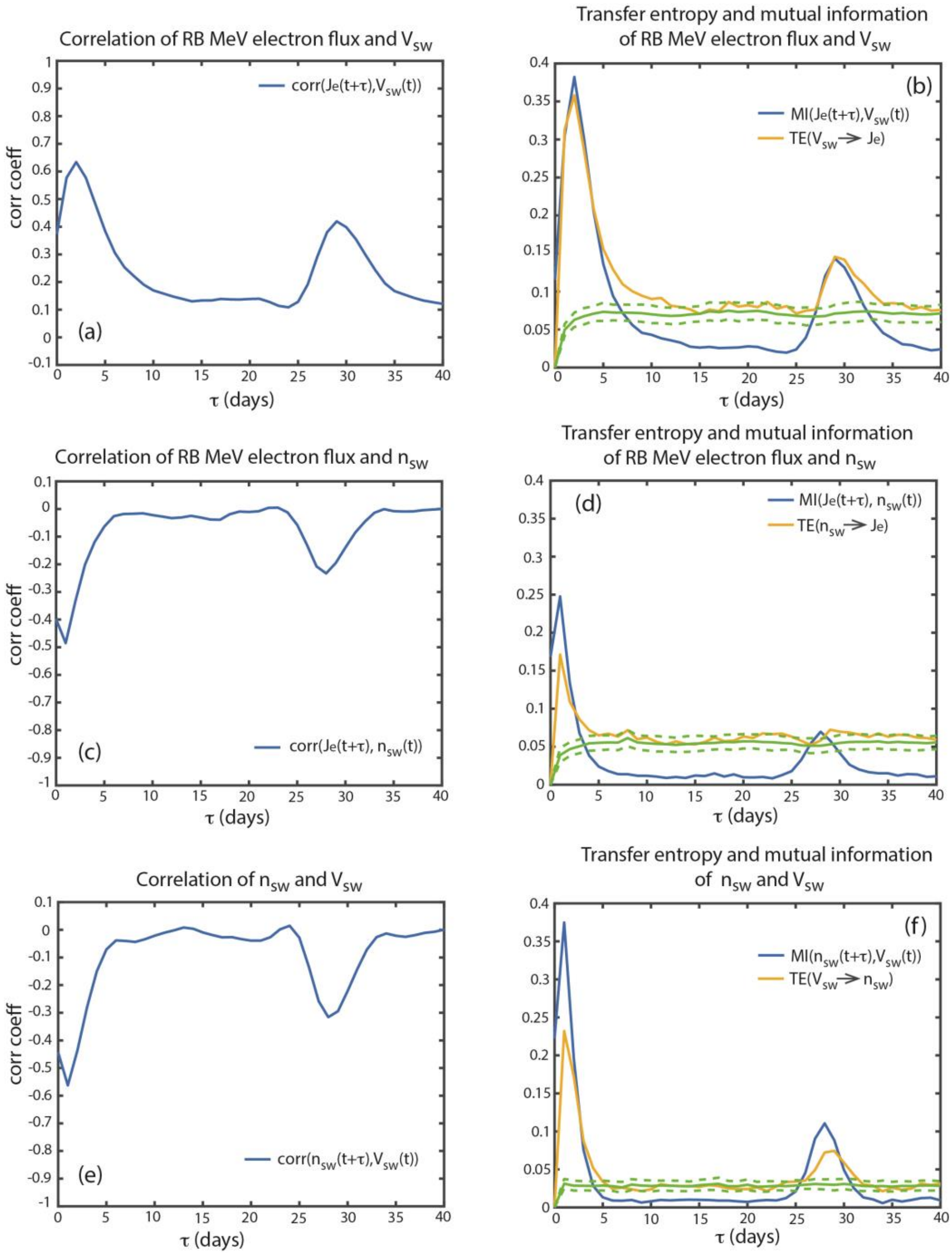

Figure 2. (a) Correlation coefficient of [$J_e(t + \tau)$, $V_{sw}(t)$]. (b) MI[$J_e(t + \tau)$, $V_{sw}(t)$] (blue) and TE[$J_e(t + \tau)$, $V_{sw}(t)$] (yellow). The transfer of information from V_{sw} to J_e [TE ($V_{sw} \rightarrow J_e$)] peaks at τ_{max} = 2 days. (c) Correlation coefficient of [$J_e(t + \tau)$, $n_{sw}(t)$]. (d) MI[$J_e(t + \tau)$, $n_{sw}(t)$] (blue) and TE[$J_e(t + \tau)$, $n_{sw}(t)$] (yellow). The transfer of information from n_{sw} to J_e [TE ($n_{sw} \rightarrow J_e$)] peaks at τ_{max} = 1 day. (e) Correlation coefficient of [$n_{sw}(t + \tau)$, $V_{sw}(t)$]. (f) MI[$n_{sw}(t + \tau)$, $V_{sw}(t)$] (blue) and TE[$n_{sw}(t + \tau)$, $V_{sw}(t)$] (yellow). The solid and dashed green curves are the mean and 3σ from the mean of the noise. The transfer of information from V_{sw} to n_{sw} [TE ($V_{sw} \rightarrow n_{sw}$)] peaks at τ_{max} = 1 day. (adapted from Wing et al., 2016.).

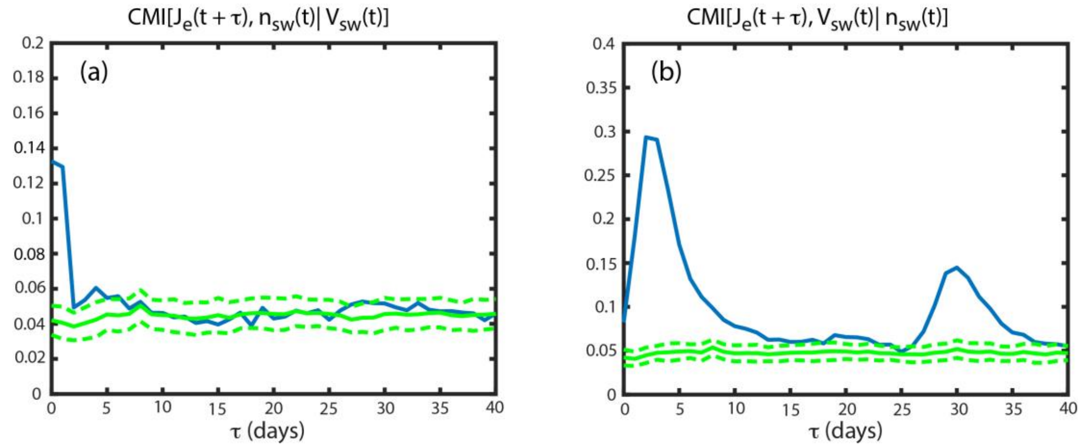

Figure 3. Blue curve showing (a) CMI[$J_e(t+\tau)$, $n_{sw}(t) \mid V_{sw}(t)$], and (b) CMI[$J_e(t+\tau)$, $V_{sw}(t) \mid n_{sw}(t)$]. The solid and dashed green curves are the mean and 3σ from the mean of the noise. (a) Unlike TE[$J_e(t+\tau)$, $n_{sw}(t)$], which peaks at τ_{max} = 1 day, CMI[$J_e(t+\tau)$, $n_{sw}(t) \mid V_{sw}(t)$] peaks at τ_{max} = 0 day (it_{max} = 0.091). The smaller τ_{max} comes about because CMI removes the effect of V_{sw} on J_e (see text). (b) The peak in CMI[$J_e(t+\tau)$, $V_{sw}(t) \mid n_{sw}(t)$] (it_{max} = 0.25) is broader and has slightly higher *snr* than that of TE[$J_e(t+\tau)$, $V_{sw}(t)$] in Figure 6.2b because CMI removes the effect of n_{sw}, which anticorrelates with J_e. V_{sw} transfers about 2.7 times more information to J_e than n_{sw}. (from Wing et al., 2016.).

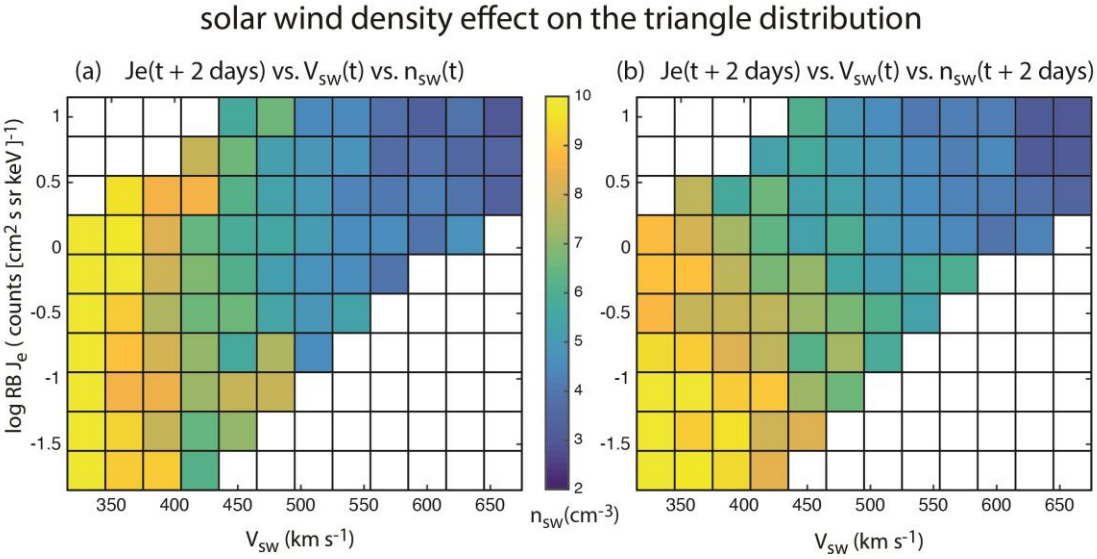

Figure 4. Points in $J_e(t + 2$ days) vs. $V_{sw}(t)$ distribution in Figure 6.10a are binned in 0.3 counts (cm^2 s sr keV)$^{-1}$ × 30 km s^{-1} bins. Each point is assigned its $n_{sw}(t)$ and $n_{sw}(t + 2$ days) values. The latter has no time shift with respect to J_e such that information transfer from n_{sw} to J_e maximizes. (a) shows the mean $n_{sw}(t)$ while (b) shows the mean $n_{sw}(t + 2$ days) of each bin. In (a), the density gradient is mainly in the x direction due to the anticorrelation between n_{sw} and V_{sw}. However, in (b), there are density gradients in x and y direction. The latter can be attributed to P_{dyn} and magnetopause shadowing. (from Wing et al., 2016.).

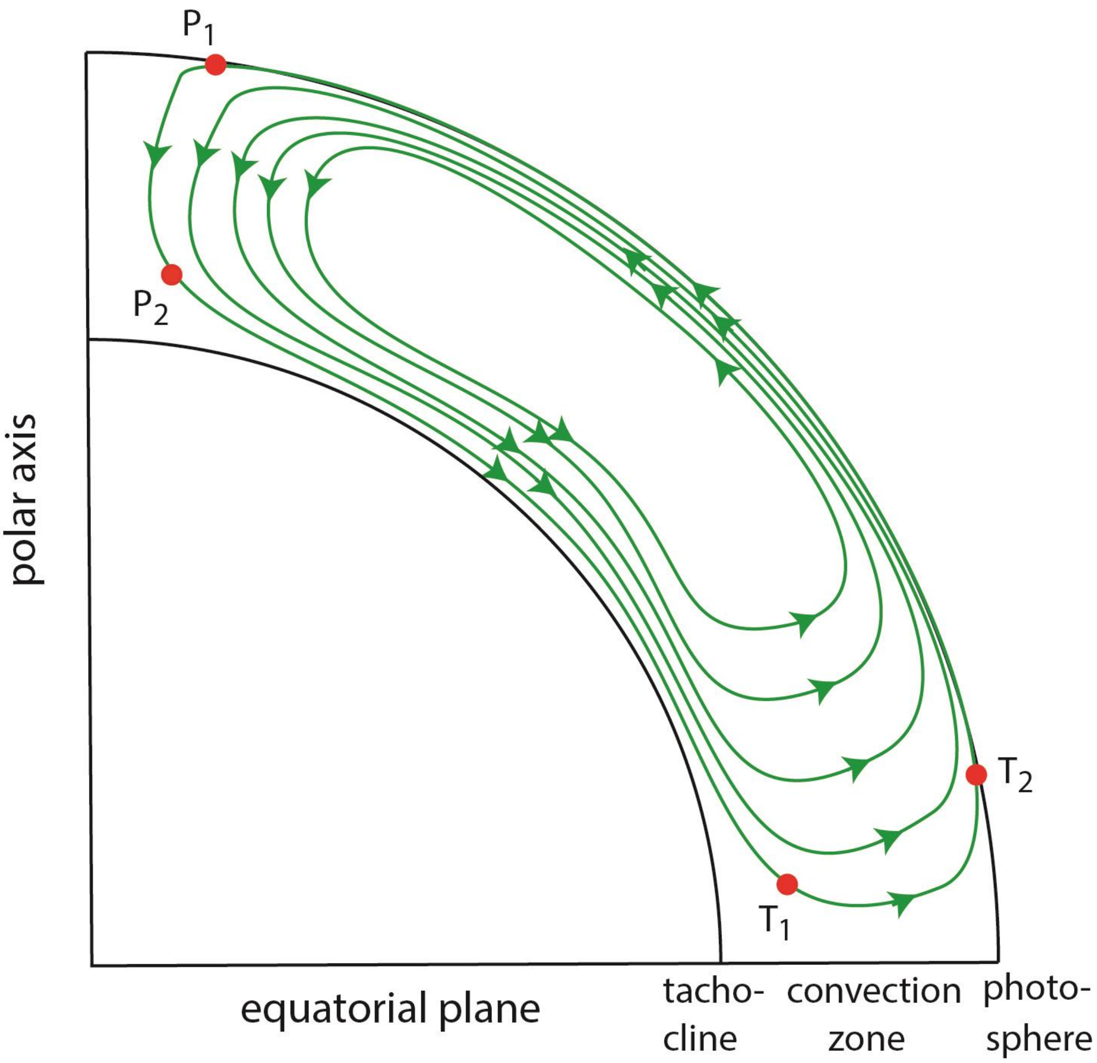

Figure 5. Babcock-Leighton type solar cycle dynamo model. The diagram shows a meridional slice of the sun. The meridional flow is plotted in green with arrows indicating the flow direction. Poloidal field at P_1 is advected down to P_2 in the convective zone by the meridional flow. The meridional flow advects the field from P_2 to T_1, while the differential rotation shears the field, converting it to toroidal field. The buoyancy force lifts the toroidal field from T_1 to the photosphere at T_2, producing sunspots. The sunspots decay into poloidal field, which is carried by the meridional flow to the T_1 and the cycle starts over again. (from Wing et al., 2018).

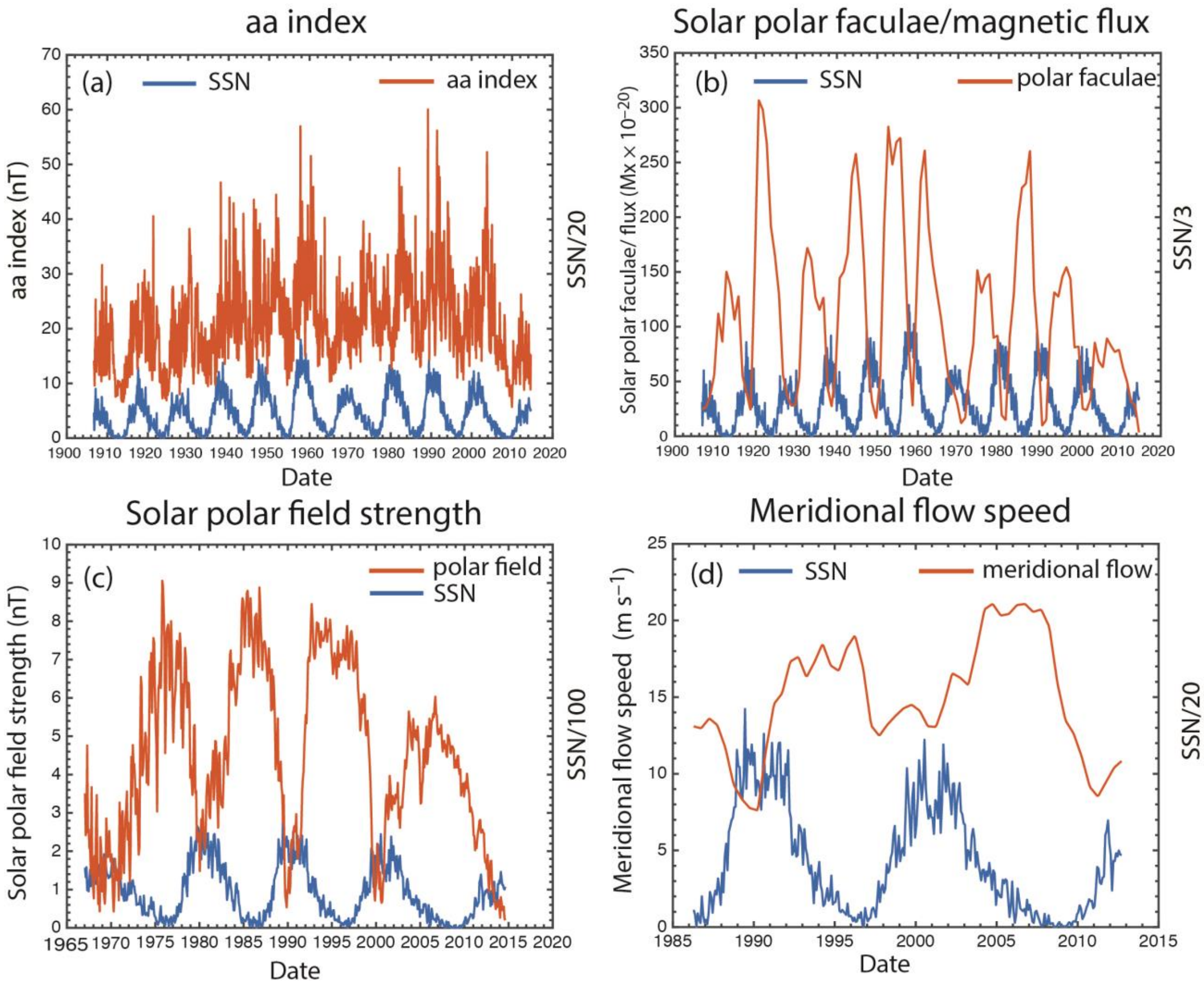

Figure 6. Solar cycle variations of (a) *aa* index; (b) the solar polar faculae calibrated to SOHO MDI polar magnetic flux (Muñoz-Jaramillo et al., 2012); (c) the solar polar field strength; (d) the meridional flow. These parameters are plotted in red curves whereas the *SSN* is plotted in the blue curves. The *SSN* has been scaled by a different factor in each figure as indicated by the right y-axis label in order to enhance viewing. (from Wing et al., 2018).

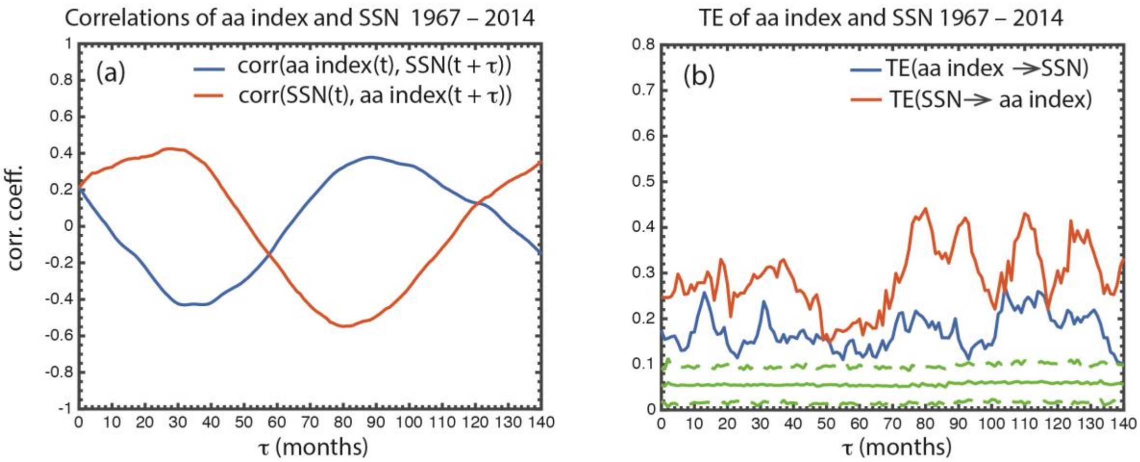

Figure 7. (a) Shifted correlation corr[*aa* index(t), *SSN*(t + τ)] is plotted in blue and corr[*SSN*(t), *aa* index(t + τ)] is plotted in red. The peak |corr[*aa* index(t), *SSN*(t + τ)]| is roughly the same as the peak |corr[*SSN*(t), *aa* index(t + τ)]|. (b) TE(*aa* index → *SSN*) is plotted in blue and TE(*SSN* → *aa* index) is plotted in red. TE(*SSN* → *aa* index) > TE(*aa* index → *SSN*), suggesting that more information is transferred from the *SSN* to *aa* index than the other way around. Such information cannot be discerned from the correlations shown in (a). The solid and dashed green curves show the mean and 3σ of the noise (see text). The data are for the period 1967–2014. (from Wing et al., 2018).

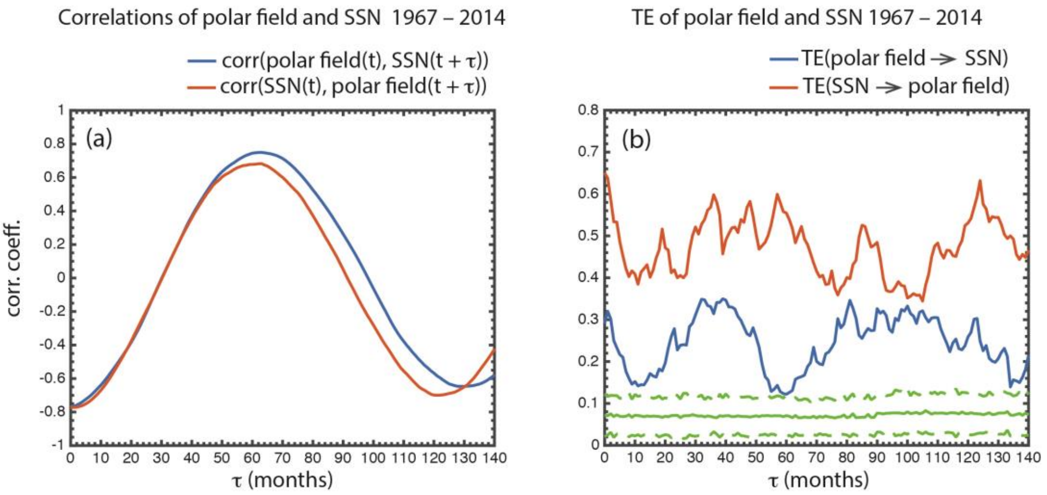

Figure 8. (a) Shifted correlation corr[polar field(t), *SSN*(t + τ)] is plotted in blue and corr[*SSN*(t), polar field(t + τ)] is plotted in red. They both reach minima at τ ~0 month and maxima at τ ~60–70 months (half solar cycle period) because the polar field and *SSN* tend to be 180° out of phase with each other. (b) TE(polar field → *SSN*) is plotted in blue and TE(*SSN* → polar field) is plotted in red. The format is the same as in Figure 3. The transfer of information from the polar field to *SSN* peaks at τ ~30–40 months. There is significant information transfer from the *SSN* to polar field as well. The solid and dashed green curves show the mean and 3σ of the noise. The data are for the period 1967–2014. (from Wing et al., 2018).

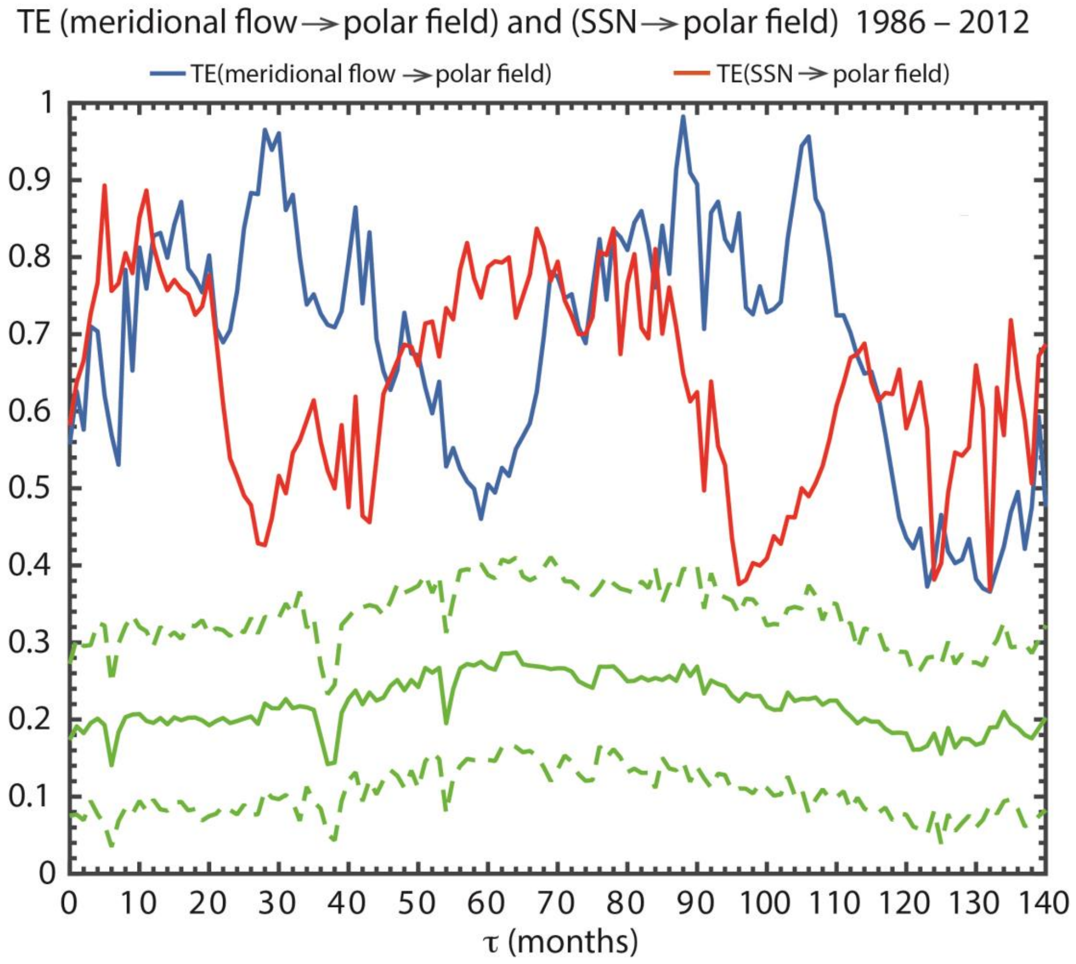

Figure 9. TE(meridional flow → polar field) and TE(*SSN* → polar field) are plotted in blue and red curves, respectively, for the period 1986–2012. The curves are noisy because of the limited availability of the meridional flow data. Both the meridional flow speed and *SSN* (proxy for flux emergence) transfer information to the polar field, but the meridional flow speed transfers more information to the polar field than *SSN* at τ ~28–30 months and τ ~90–110 months. On the other hand, the *SSN* transfers more information to the polar field than the meridional flow at τ ~60–80 months. The solid and dashed green curves show the mean and 3σ of the noise. (from Wing et al., 2018).

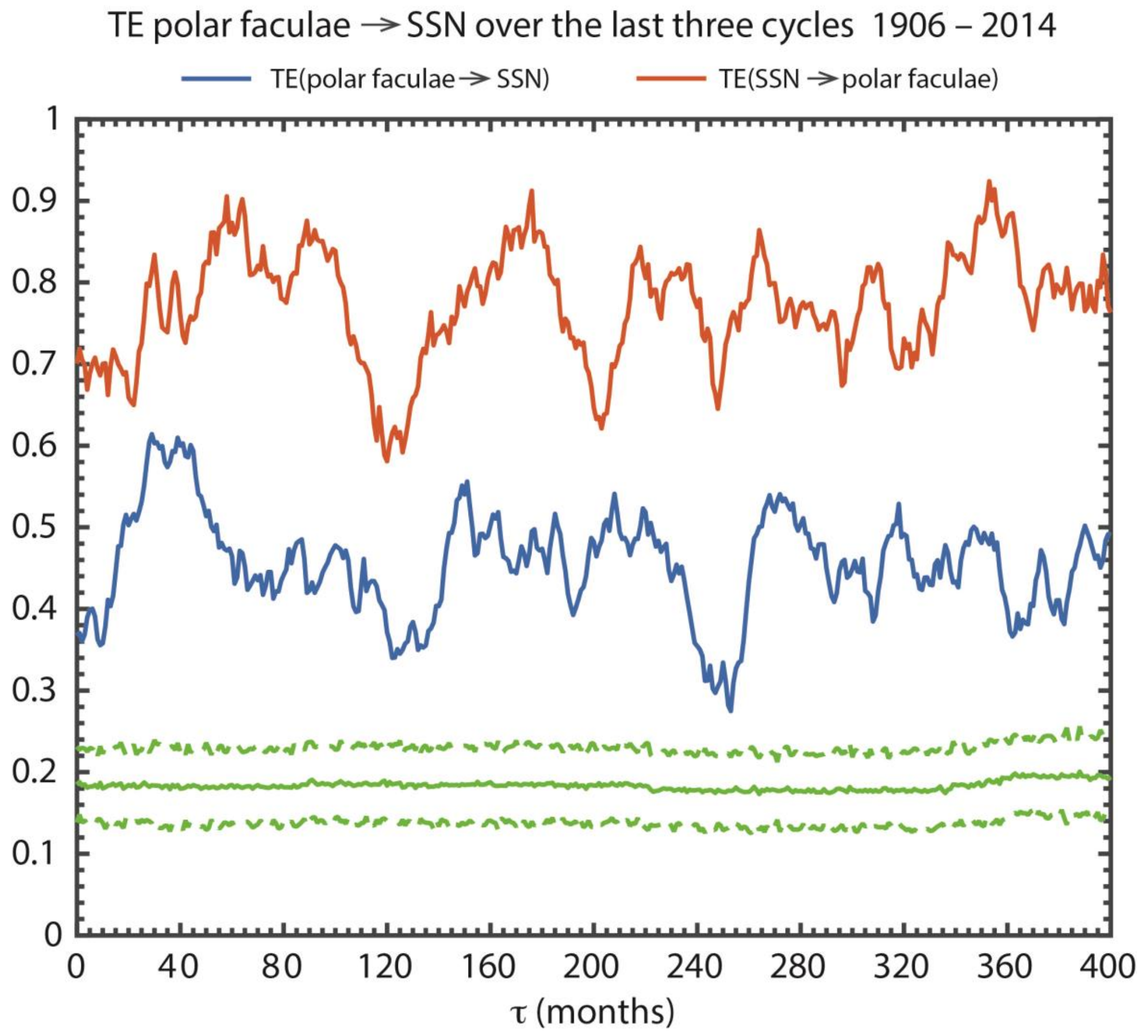

Figure 10. The long term effect of the polar fields (as proxied by the polar faculae) on sunspot production. TE(polar faculae → *SSN*) and TE(*SSN* → polar faculae) are plotted in blue and red curves, respectively, for the period 1906–2014. The transfer of information from the polar faculae (proxy for the polar fields) to *SSN* peaks at τ ~30–40 months, but thereafter it persists for at least 400 months (~3 solar cycle period) albeit at lower level. The solid and dashed green curves show the mean and 3σ of the noise. There is also a long term effect of the *SSN* on polar faculae. (from Wing et al., 2018).
